# Multiscale Electrochemistry of Lithium Manganese Oxide (LiMn_2_O_4_): From Single Particles to Ensembles and Degrees of Electrolyte Wetting

**DOI:** 10.1021/acssuschemeng.2c06075

**Published:** 2023-01-13

**Authors:** Binglin Tao, Ian J. McPherson, Enrico Daviddi, Cameron L. Bentley, Patrick R. Unwin

**Affiliations:** † Department of Chemistry, University of Warwick, Coventry CV4 7AL, United Kingdom; ‡ School of Chemistry, Monash University, Clayton 3800, VIC, Australia

**Keywords:** Li^+^ transfer kinetics, contact resistance, ensemble effect, scanning electrochemical cell microscopy, LiMn_2_O_4_ particles

## Abstract

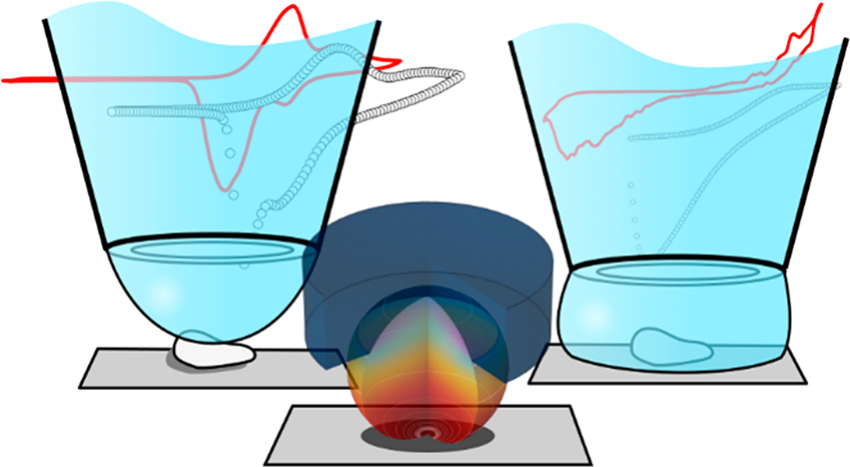

Scanning electrochemical cell microscopy (SECCM) facilitates single particle measurements of battery materials using voltammetry at fast scan rates (1 V s^–1^), providing detailed insight into intrinsic particle kinetics, otherwise obscured by matrix effects. Here, we elucidate the electrochemistry of lithium manganese oxide (LiMn_2_O_4_) particles, using a series of SECCM probes of graded size to determine the evolution of electrochemical characteristics from the single particle to ensemble level. Nanometer scale control over the SECCM meniscus cell position and height further allows the study of variable particle/substrate electrolyte wetting, including comparison of fully wetted particles (where contact is also made with the underlying glassy carbon substrate electrode) vs partly wetted particles. We find ensembles of LiMn_2_O_4_ particles show voltammograms with much larger peak separations than those of single particles. In addition, if the SECCM meniscus is brought into contact with the substrate electrode, such that the particle–support contact changes from *dry* to *wet*, a further dramatic increase in peak separation is observed. Finite element method modeling of the system reveals the importance of finite electronic conductivity of the particles, contact resistance, surface kinetics, particle size, and contact area with the electrode surface in determining the voltammetric waveshape at fast scan rates, while the responses are relatively insensitive to Li^+^ diffusion coefficients over a range of typical values. The simulation results explain the variability in voltammetric responses seen at the single particle level and reveal some of the key factors responsible for the evolution of the response, from ensemble, contact, and wetting perspectives. The variables and considerations explored herein are applicable to any single entity (nanoscale) electrochemical study involving low conductivity materials and should serve as a useful guide for further investigations of this type. Overall, this study highlights the potential of multiscale measurements, where wetting, electronic contact, and ionic contact can be varied independently, to inform the design of practical composite electrodes.

## Abstract 2

Scanning electrochemical cell microscopy and modeling probe particle kinetics, conductivity, electrolyte wetting, and ion diffusion in one high-throughput measurement.

## Introduction

The high power demands of modern electric vehicles have driven extensive research into improving the power density (rate capability) of Li-ion batteries.^1,2^ Focusing on the positive electrode, among a host of different metal oxide materials, lithium manganese oxide (LiMn_2_O_4_) spinel is widely used due to its large theoretical energy capacity, the relatively high abundance of Mn, and its relatively low environmental impact.^3−5^ While it is reported that the overall rate capability may be limited by charge-transfer (i.e., ion and/or electron transport) into the LiMn_2_O_4_ particles themselves,^6^ few studies explicitly consider the electrochemical properties of particles in the absence of “matrix effects”.^7,8^ This is because in practice it is difficult to isolate the response of “a particle” within a complex composite electrode (i.e., active material, binder, conductive agent, and current collector), where the influence of contact resistance between the active particles and/or current collector,^9,10^ variable wetting of particles,^11,12^ and interparticle variations in electrode structure^13^ may all contribute to the macroscopic electrochemical response.^8^ Thus, there is a great need for new techniques to study battery electrode materials in a systematic way, initially by removing all of the auxiliary elements and focusing only on the electrochemistry of single LiMn_2_O_4_ particles or small ensembles of active particles.^14^

Microelectrode^15−19^ and even nanoelectrode^20^ techniques have been exploited as effective methodologies to investigate the electrochemical properties of single particle electrodes. The beauty of such approaches is that they avoid the need for the binders and conductive additives used in the fabrication of macroscopic composite electrodes, thus allowing the intrinsic properties of active materials (particles) to be revealed. For example, single particles measured in the microelectrode format exhibit much higher rate capabilities, with apparent Li^+^ ion diffusion coefficients that are 2 to 5 orders of magnitude higher compared to that measured in bulk.^16^ Based on this study and others,^15,20^ it has been deduced that future research regarding the rate performance of batteries should focus on the electrode design/architecture (e.g., the electrical/ionic connectivity of particles).^7,18,21^ Nevertheless, single particle analysis by way of microelectrodes and nanoelectrodes is inherently low throughput (i.e., single particle at a time) and only offers limited insight into the variation present within a population of particles.^8^

Scanning electrochemical cell microscopy (SECCM) has emerged as a powerful technique for probing electrochemistry down to the single-entity level for a wide variety of electrode materials, including those used in batteries, fuel cells, and electrolyzers.^14,22−32^ In SECCM, a fluidic micropipette/nanopipette probe is used to perform electrochemistry within a confined area of an electrode surface, with spatial resolution defined by the area of meniscus (ionic) contact.^33−35^ In contrast to the microelectrode techniques mentioned above, SECCM targets the electrochemistry of a series of individual particles/clusters in a single high-throughput scanning experiment, which can be further correlated to colocated structural information to assign single particle structure–activity unambiguously.

Single particle techniques expand the utility of voltammetric methods in battery research, which are hampered by the considerable resistive and capacitive contributions of the associated matrix components when operated in macroscale measurements. The small (ionic) contact area of SECCM leads to very low overall currents, free from ohmic effects in the electrolyte, permitting voltammetry with fast scan rates.^29,36^ Not only does this increase the throughput of SECCM measurements, but faster scan rates provide an increased dynamic window into the kinetics of particles, which is important when fast-charging battery materials are studied. For example, our group recently used this approach to interrogate single LiMn_2_O_4_ particles isolated from an ensemble, revealing superfast charge/discharge rate capability (up to 279 C) at the single particle level.^14^ The study also revealed significant variations in waveshapes between particles, and this has prompted us to examine voltammetry at the single particle level in more detail in this paper.

The theory of Li^+^ intercalation in battery materials is well established^37,38^ and has been applied to single particle voltammetry previously;^39−41^ however, a number of simplifying assumptions around particle shape, conductivity, and particle–substrate contact are often used. Such assumptions may be valid for close-to-equilibrium measurements, such as slow scan rate voltammetry, where information on these properties is lost. In contrast, SECCM at fast scan rates becomes sensitive to these properties, although interpretation is necessarily more involved. For example, while current peak position, or positive/negative peak separation (Δ*E*_p_), is generally used as an indicator of electrode kinetics (slower kinetics require a greater deviation from equilibrium to reach the same peak current), a significant series resistance (e.g., low intraparticle conductivity, particle/substrate contact resistance) will also lead to increased Δ*E*_p_.^42^ More specifically, understanding the voltage losses that occur along a current path is crucial to probe the intrinsic electron transfer rates of individual interfaces.^43−45^

Here, we build on our previous study to investigate the voltammetry of LiMn_2_O_4_ particles in more detail. We study the evolution in voltammetric waveshape from individual LiMn_2_O_4_ particles (the limiting case of a “dilute electrode”)^21^ to clusters of ca. 5–10 particles then to the ensemble level (ca. 100 particles) by utilization of a series of micropipette probes with graded diameters. Precisely controlling the position of the micropipette in 3D space allows both the number of LiMn_2_O_4_ particles, as well as the wetting of the supporting electrode, to be controlled, permitting chemical (ensemble) and physical (particle conductivity, particle/substrate contact resistance) effects to be deconvoluted. We then use complementary finite element method simulations to interpret the voltammetry in terms of particle size, state of charge-dependent conductivity, and extent of particle–substrate contact and particle/substrate wetting.

## Experimental Section

### Chemical Reagents and Electrodes Preparation

Lithium manganese oxide (LiMn_2_O_4_, spinel structure, <0.5 μm particle size) and lithium chloride (LiCl, ≥99%) were both purchased from Sigma-Aldrich and used as received. Deionized water (resistivity ≥18 MΩ·cm) was produced by a Purite Integra HP system (U.K.). The glassy carbon (GC) plate (25 mm × 25 mm) was purchased from Alfa Aesar and cleaned with 0.05 μm Al_2_O_3_ suspension (Buehler, U.S.A.) prior to use. Highly oriented pyrolytic graphite (HOPG) was purchased from SPI Materials (12 mm × 12 mm × 2 mm). Before use, HOPG was cleaved to expose a fresh surface using scotch tape. To prepare the working electrodes, LiMn_2_O_4_ particles were sonicated in deionized water for 10 min and left to stand at room temperature for 30 min, and then, 0.6 μL of the supernatant was drop-cast onto different carbon substrates. After drying under ambient conditions (ca. 30 min), these electrodes were mounted on an *xy* piezoelectric positioner for SECCM (*vide infra*). The silver/silver chloride (Ag/AgCl) quasi-reference counter electrode (QRCE) was prepared by positive polarization of an Ag wire (0.125 mm diameter, Goodfellow, 99.99%) at 5 V vs Pt wire in a saturated KCl solution. The QRCE potential was calibrated against a commercial saturated calomel electrode (SCE) in a 1 M LiCl solution, which possessed a stable reference potential of ±0.005 V vs SCE.

### Instrumentation

Single channel micropipettes with diameters of 2 and 5 μm were prepared using a CO_2_ laser puller (P-2000, Sutter Instruments, U.S.A.). The former were pulled from glass capillaries (GC 120F-10, 1.2 mm outer diameter × 0.69 mm inner diameter × 100 mm length, Harvard Apparatus, U.S.A.) with a one-step protocol. The parameters were heat 350, filament 4, velocity 40, delay 200, and pull 0. The latter were pulled from quartz capillaries (QTF 120-90-100, Friedrich & Dimmock, Inc., U.S.A.), and another one-step protocol was exploited. The parameters were heat 680, filament 4, velocity 45, delay 130, and pull 35. The dimensions of the micropipette orifice were measured using scanning transmission electron microscopy (STEM) on a Zeiss Gemini 500 system, which was operated at an accelerating voltage of 10 kV. This system was also used for the observation of LiMn_2_O_4_ particle morphology via switching to the scanning electron microscopy (SEM) mode. Single channel micropipettes with diameters of 70 μm were pulled from glass capillaries (GC 120F-10, 1.0 mm outer diameter × 0.58 mm inner diameter × 100 mm length, Harvard Apparatus, U.S.A.) using a PC-10 puller (Narishige Group, Japan) with a two-step protocol. For the first step, the parameters were heat 65, weight 3, and slider 8. For the second step, the parameters were heat 55, weight 3, and slider 4. The diameter of the tip was measured with an optical microscope (BH-2 optical microscope, Olympus, Japan). After pulling, these micropipette probes were filled with a 1 M LiCl solution using a MicroFil syringe (World Precision Instrument, Inc., U.S.A.) and a lab-made Ag/AgCl QRCE (mentioned above) was inserted from the back of the capillary.^46^

All experiments were performed on a custom-made scanning electrochemical cell microscopy (SECCM) platform unless specified, which was placed on automatic leveling isolators (Newport, S-2000A-423.5) to minimize vibration. The micropipette probe was mounted on a *z* piezoelectric positioner (P-753.3CD, Physik Instrumente, Germany), controlled by an amplifier module (E-665), for positioning normal to the sample. Movement of the working electrode (sample) in the horizontal plane was controlled by an *xy* piezoelectric positioner (P-622.2CD, Physik Instrumente), with E-500 amplifier modules.

Typically, the voltage was biased on the Ag/AgCl QRCE in the micropipette, with respect to a common ground, and the current on the working electrode, at ground, was measured via a custom-made current follower (different sensitivities in different experiments, *vide infra*). It should be noted that all the piezoelectric positioners and the electrometer head were placed inside an aluminum Faraday cage. During experiments, the surface current was measured every 4 μs, which was averaged 512 times to give a data acquisition rate of 4 × (512 + 1) = 2052 μs (note that one extra iteration is used to transfer the data to the host computer). To achieve such fast signal sending and data acquisition/processing capability, a field programmable gate array (FPGA) board (PCIe-7852R) from NI (National Instruments, U.S.A.) company was exploited. The whole system was controlled by a LabVIEW 2019 interface running the Warwick electrochemical scanning probe microscopy software (WEC-SPM, www.warwick.ac.uk/electrochemistry).

### Scanning Protocol

For the experiments with small micropipettes (2 and 5 μm), the scanning protocol was exactly the same as our previous report^14^ (scan *voltammetric hopping mode*). Briefly, the micropipette (meniscus cell) was approached to the surface of interest at a speed of 3 μm s^–1^, during which the Ag/AgCl QRCE was biased at −1.25 V (i.e., working electrode potential of +1.25 V with respect to Ag/AgCl QRCE), and the current at the working electrode was monitored constantly. Upon meniscus landing, i.e., when an electrochemical cell was formed between the micropipette and sample surface through the meniscus, the electric circuit was closed, and a threshold current of 2 pA (slightly larger than the system noise level of ±1.3 pA at a current range of ±1 nA) triggered the tip to stop from further approaching. The ability to use very low threshold currents to detect meniscus contact resulted in a range of wetting conditions, depending on the exact particle geometry, that could be explored and analyzed (*vide infra*). The working electrode potential switched immediately to 0 V, and a local cyclic voltammetric experiment was performed (from 0 to 1.25 V then back to 0 V vs Ag/AgCl QRCE), following which the micropipette was retracted 5 or 10 μm from the surface (depending on the tip size). The micropipette was subsequently moved to the next predefined pixel, located 5 or 10 μm from the previous point, set by the predefined “hopping distance”. At each and every pixel, the electrochemical signals (potential, *E*, and current, *i*) were recorded, but only the active pixels (with signals different from bare substrate) were analyzed in this work.

Different from the scanning protocol mentioned above, experiments performed with the larger tips (diameter of 70 μm) were single point based, via a *step-approach cyclic voltammetry*. The micropipette (meniscus cell) was translated toward the surface of interest at a speed of 0.5 μm s^–1^, during which the Ag/AgCl QRCE was again biased at −1.25 V, and the current on the working electrode was monitored constantly. A threshold current of 50 pA (system noise level of ±5 pA, at a current range of ±10 nA) triggered the tip to stop approaching. At this height, a local cyclic voltammetric experiment was performed (0 to 1.25 V vs Ag/AgCl QRCE), with the vertical extension of the micropipette (*z* coordinate) and electrochemical signals (potential, *E*, and current, *i*) recorded. The tip was then moved down a specific distance of 100 nm using the *z* piezoelectric positioner, and another local cyclic voltammetric experiment was performed. By repeating these steps of moving down and performing a cyclic voltammetric measurement several times, a series of CVs can be recorded, which gave information at increasing degrees of wetting until the carbon-based substrate was touched.

### Data Processing

Collected data were processed with Matlab R2015b to extract the active pixels in each experiment. Data plotting was performed using Matlab R2015b and Origin 2018 software packages. It should be noted that there is no interpolation or smoothing of any SECCM data presented in this work. All the SEM figures were cropped and typeset with Adobe Photoshop 2017.

## Results and Discussion

### Experimental Design and Multiscale Analysis of LiMn_2_O_4_ Particles

**Figure 1:**
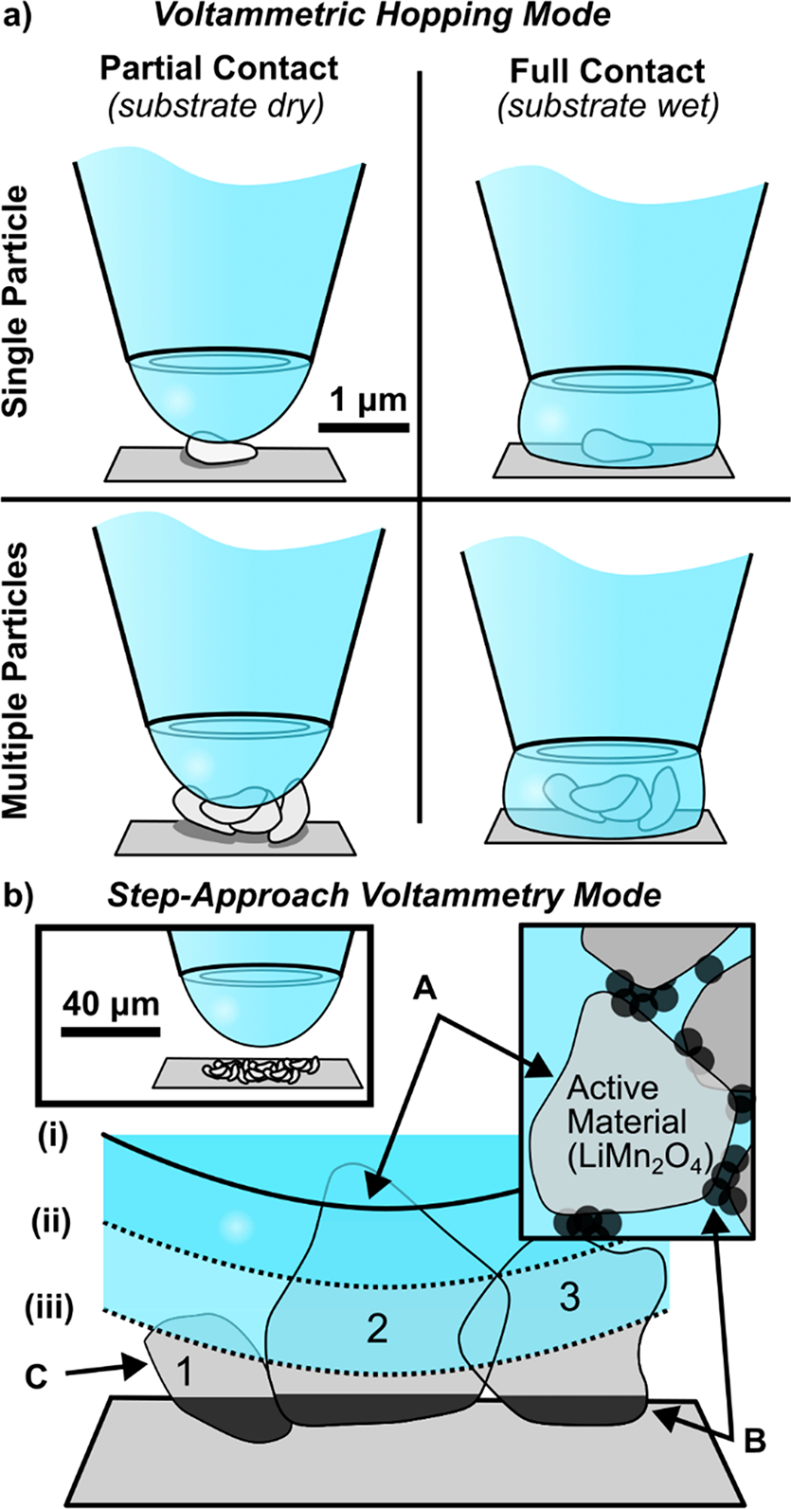
Two types of SECCM experiments used in this work. (a) In *voltammetric hopping mode*, four types of meniscus contacts with the particles are made, depending on the extent of wetting and number of particles encountered at each pixel of the scan area. (b) In s*tep-approach cyclic voltammetry mode*, a larger micropipette is used to sample a larger number of particles (left inset) and is incrementally moved downward between voltammograms to (ionically) contact more and more particles [e.g., from position (i) to position (iii)]. The correspondences between ionic (A) and electronic (B) contact areas in this measurement and in a composite battery electrode are illustrated (right inset), with SECCM also having an uncontacted area (C).

Two different types of experiments were designed (Figure 1). In the first experiment, SECCM was deployed in the *voltammetric hopping mode* to interrogate the redox activity of a series of individual LiMn_2_O_4_ particles or particle clusters (<10 particles) supported on GC, as detailed in our previous study.^14^ After completing SECCM scans, the areas were visualized with scanning electron microscopy (SEM), and each individual cyclic voltammogram (CV) was classified according to the number of particles contacted (i.e., *single* or *multiple*) and the nature of the meniscus contact (i.e., *partial* contact with the top of the particle only, leaving the substrate dry and free of residue, versus *full* contact with the particle, including wetting the substrate, and leaving a circular region of brighter contrast in SEM, Figure 1a).

In the second series of experiments, a relatively large micropipette probe (diameter ≈70 μm) was employed to perform *step-approach cyclic voltammetry* on small ensembles of LiMn_2_O_4_ particles (ca. 15 agglomerates or 100 particles). In this type of experiment, the micropipette was halted upon making initial meniscus contact with only the tops of the LiMn_2_O_4_ particles (e.g., meniscus position (i) in Figure 1b; details in the Experimental Section,) and then, a CV was recorded. The probe was then translated 100 nm down, toward the support, and another CV recorded (e.g., meniscus position (ii) in Figure 1b). This incremental translation and CV measurement were repeated until the meniscus wetted the GC support (as determined from the change in CV behavior, *vide infra*).

In macroscopic experiments, where (neglecting the role of the binder) the active material is either in ionic contact with the electrolyte (‘A’ in Figure 1b) or electronic contact with the conductive additive (‘B’ in Figure 1b), it is not possible to independently vary each to examine their effect (Figure 1b, right inset). In contrast, the “cross-sectional” approach to SECCM allows the electrolyte (ionic) contact to be varied independently of conductor contact (leaving area ‘C’ in Figure 1b uncontacted), allowing different extents of the electrolyte–particle–substrate interaction to be examined.

In *step-approach voltammetry*, with a large probe, initial meniscus contact results in wetting of a low density ensemble of particles with a dry particle–support contact, with only the upper parts of the larger LiMn_2_O_4_ particle(s) contacted by the meniscus (meniscus (i) in Figure 1b, so only particle 2 is contacted), while the lower parts of these particles, as well as many other small particles (e.g., particles 1 and 3 in Figure 1b), remain dry. Upon further translation of the probe toward the surface, an intermediate density ensemble of particles, still with a dry particle–support contact, is achieved (meniscus (ii), Figure 1b). By comparing CVs obtained from the low and intermediate density ensembles, the influence of interparticle interactions (i.e., ensemble effects) on Li^+^ (de)intercalation at LiMn_2_O_4_ can be inferred. As the probe is translated still further (to point iii and beyond), the meniscus eventually wets the substrate and the complete ensemble of particles. Now, comparing CVs of the intermediate density ensemble, where the substrate was not wetted, with the fully wetted ensemble and substrate, the role of the particle–support interaction (i.e., *wet* vs *dry* contact) in modulating Li^+^ (de)intercalation rates at LiMn_2_O_4_ can be inferred. In effect, the experiments probe the LiMn_2_O_4_ electrode across length scales, from the single particle to the ensemble (ca. 100 particles) level to identify the multiscale factors controlling the voltammetry of Li^+^ (de)intercalation

### Li^+^ (De)intercalation at 1–10 LiMn_2_O_4_ Particles

**Figure 2:**
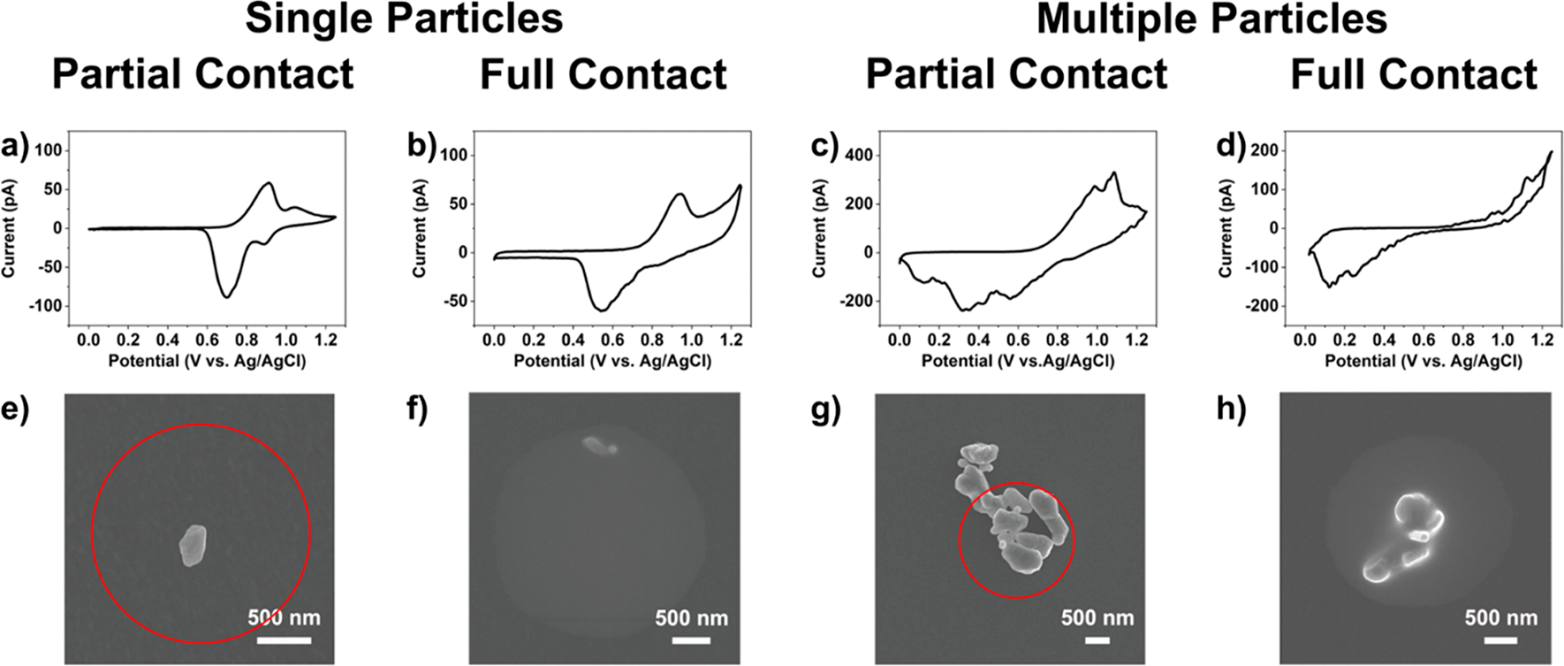
CVs (a–d) and corresponding postscan SEM images (e–h) from representative LiMn_2_O_4_ particle(s) supported on GC with differing extents of meniscus contact. The CV measurements (ν = 1 V s^–1^) were obtained with probes of diameters ca. 2 μm filled with 1 M LiCl solution. For partial contact, the red circles indicate the estimated meniscus position based on neighboring full contact cases, knowing the precise pipet probe locations in the *xy* plane, measured in the SECCM scanning protocol with nm precision.

Employing the experimental protocol outlined in Figure 1a, *voltammetric hopping mode* SECCM was performed on GC-supported LiMn_2_O_4_ particles, initially using a micropipette probe of a diameter ca. 2 μm (representative image is shown in the Supporting Information, SI, Section 1, Figure S1a). Representative CVs obtained for the four different contact modes are shown in Figure 2. In the first case (*single* particle, *partial* meniscus contact) intercalation occurs only from the upper portion of the LiMn_2_O_4_ particle wetted by the meniscus, while the GC substrate and the dry particle–support contact simply served as series resistance in the electrical circuit. The two pairs of redox peaks located at 0.90/0.70 V and 1.05/0.89 V correspond to two different Li^+^ extraction–insertion processes, which can be assigned to Li^+^ extraction from tetrahedral lattice sites in the presence and absence of the Li–Li interaction, respectively.^3^ At this scan rate (ν = 1 V s^–1^), the first pair of peaks show significantly higher peak currents than the second pair, in contrast to previous single particle observations at much slower scan rates.^15^ Further, the scan rate is 2–4 orders of magnitude larger than that typically employed in bulk measurements (0.1–10 mV s^–1^) with the same material in a composite electrode.^47,48^ It is important to note that while the CV shown in Figure 2a is considered representative, each individual LiMn_2_O_4_ particle presents a unique *i–E* characteristic, as presented in the SI, Section 1, Figure S2a–f.

In the second case (*single* particle, *full* contact), both the single LiMn_2_O_4_ particle and the GC support underwent electrochemical reactions, and the resulting CV is the sum of the two processes. Consulting Figure 2b, the pair of peaks located at 0.94/0.55 V corresponds to Li^+^ (de)intercalation at LiMn_2_O_4_, while the process at >1 V arises from oxidation of the GC support, which effectively serves as an *in situ* indicator of whether the meniscus wetted the support or not. In this context, the signal from the GC support can be viewed as a “parasitic process” that does not interfere with the Li^+^ (de)intercalation process at LiMn_2_O_4_ (*vide infra*). It is interesting to note that the CV only features a single pair of redox peaks, with a larger Δ*E*_p_ value than seen for the *single* particle, *partial* contact case in Figure 2a. This could be a result of slower electrode kinetics, or more likely, could arise from the contact resistance brought on by the different nature of the particle–support contact, i.e., *dry* vs *wet* contact, as discussed in detail below. Other data with single particles and full contact are shown in the SI, Section 1, Figure S3a and b.

In the third case (*multiple* particles, *partial* contact), several LiMn_2_O_4_ particles (5–6 particles shown in Figure 2c) contribute to the electrochemical signal, with the CV being the sum of the individual particle responses. This is clear from the multiple overlapping peaks present in the CV, e.g., positive peaks at 0.99, 1.09, and 1.2 V and negative peaks at 0.56, 0.35, and 0.12 V on the forward and reverse sweeps, respectively. Interestingly, this leads to much broader peaks and larger Δ*E*_p_ values than single particles for either contact mode (Figure 2a, b). Again, this indicates either less-facile Li^+^ (de)intercalation kinetics at the multiple particle level or greater resistance. Other data for multiple particles with partial meniscus contact are shown in SI, Section 1, Figures S4–6.

Finally, shown in Figure 2d is a CV of multiple particles with full meniscus contact. Unlike the previous cases in Figure 2a–c, in this case, the positive processes from LiMn_2_O_4_ (i.e., Li^+^ deintercalation) overlap with the background signal (i.e., parasitic reactions) from the GC support, meaning that only negative peaks are discernible. This indicates that the system resistance has increased significantly from the partial contact case, even though the number of probed particles (5 particles) is the same.

**Figure 3:**
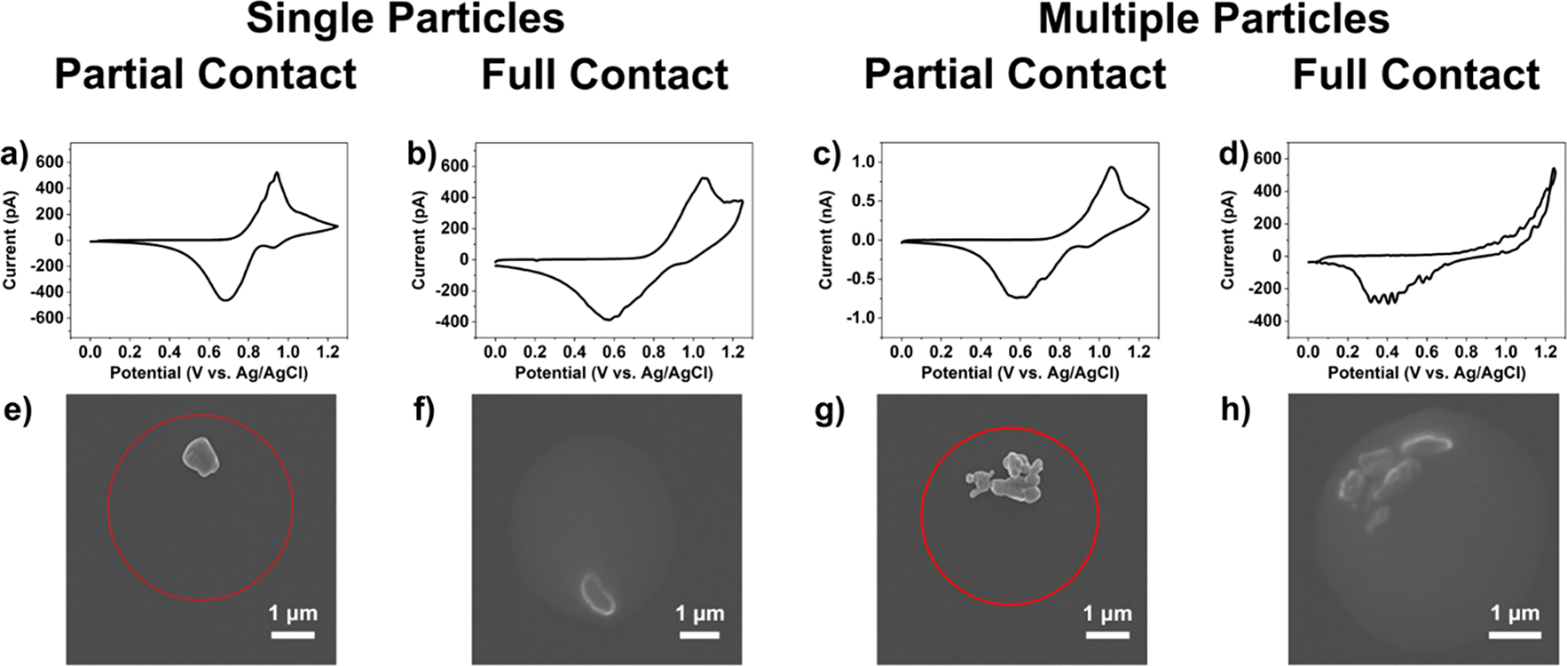
CVs (a–d) and corresponding postscan SEM images (e–h) from representative LiMn_2_O_4_ particle(s) supported on GC with differing extents of meniscus contact. The CV measurements (ν = 1 V s^–1^) were obtained with probes of diameter ca. 5 μm filled with 1 M LiCl solution.

The experiments described in Figure 1 were repeated with a larger micropipette probe (diameter ≈ 5 μm, see SI, Section 1, Figure S1b), and results are shown in Figure 3. Increasing the size of the micropipette probe has two important consequences: (i) GC makes up a larger proportion of the probed area during full contact experiments, and (ii) a larger number of particles can be simultaneously probed during multiple particle experiments.

For *single* particles with *partial* meniscus contact, Figure 3a, two pairs of redox peaks are again discernible in the CV at 0.94/0.68 and 1.06/0.93 V, attributable to two-stage Li^+^ (de)intercalation at LiMn_2_O_4_, described above. Compared with the single particle example shown in Figure 2a, the particle size in Figure 3a is ca. 2–3 times larger in diameter, explaining the higher current magnitude (ca. 8-fold different). Significantly, particle size appears to have a negligible effect on the Δ*E*_p_ values (compare Figure 2a and Figure 3a), consistent with our previous report.^14^ One interpretation is that particle resistance (increasing with particle size) does not contribute significantly to the Δ*E*_p_ value, although it is possible that the effect of increased intraparticle resistance could be offset by a larger wetted particle area (*vide infra*).

*Single* particles in *full* contact with the larger meniscus again only show one pair of redox peaks in the CV at 1.05/0.57 V, superimposed on the background (parasitic) current from the GC support (Figure 3b). *Multiple* (ca. 10) particles in *partial* contact with the larger meniscus exhibit a merged oxidation peak at 1.06 V (Li^+^ deintercalation) in the forward sweep and a series of reduction peaks at 0.57, 0.62, and 0.73 V in the reverse sweep (Li^+^ intercalation). In contrast again, multiple particles in full contact mode (Figure 3d) show very large peak shifts, with no discernible oxidation peak and a broad, drawn-out reduction peak at ca. 0.4 V. In general, the trends in Δ*E*_p_ value in Figure 3 agree with the smaller-probe counterparts in Figure 2. It should also be re-emphasized that all data depicted here are considered representative; the full collection of correlative electrochemistry–SEM measurements is available in SI, Section 1, Figures S2–S9.

**Figure 4:**
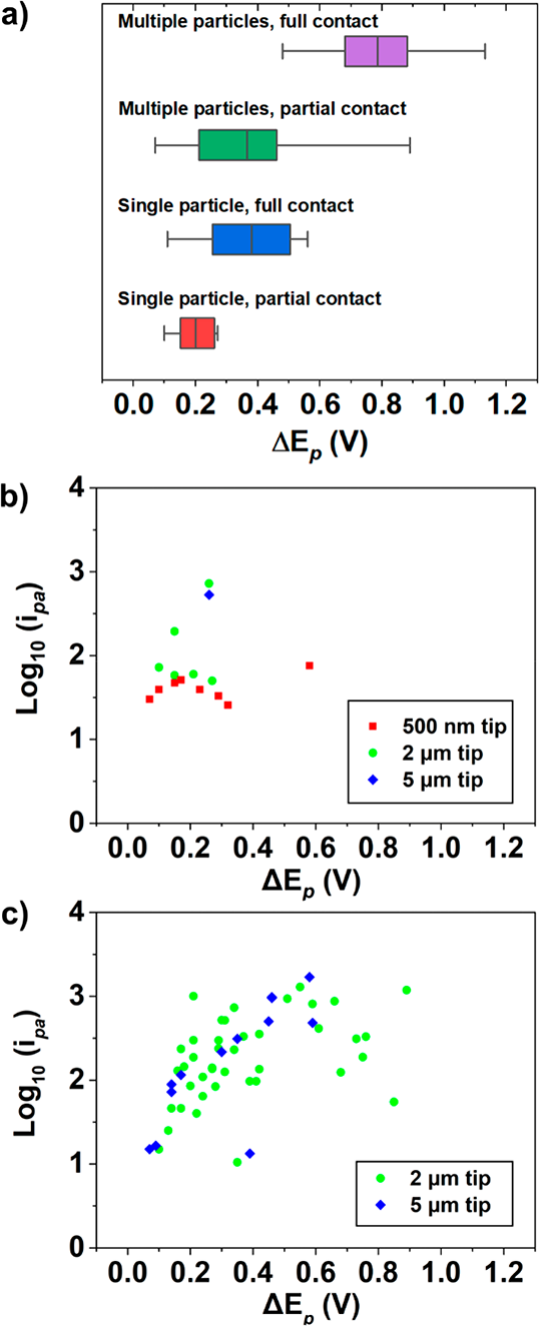
(a) Summary of peak separations with different electrolyte–particle(s)–glassy carbon contact modes; partial contact indicates only partial wetting of the particles (dry particle electrode contact) and full contact indicates full ionic wetting of the particle and support electrode. The box represents the interquartile range, the midline the mean, and the whiskers minimum and maximum values. (b) Log positive current as a function of peak separation for single particle contacts. (c) as (b) for multiparticle contacts. Data from Table S1, except 500 nm tip data.^14^

To understand how interparticle interactions (e.g., *single* vs *multiple* particles) and the particle–support contact (e.g., *wet* vs *dry* contact) may influence apparent Li^+^ (de)intercalation kinetics at LiMn_2_O_4_ (assuming no contribution from resistance),^42^ a plot of Δ*E*_p_ values (tabulated in SI, Section 1, Table S1) vs contact mode (Figure 1a) was constructed, as shown in Figure 4a–c. Evidently, Δ*E*_p_ increases in the order of meniscus contact mode: single particle, partial contact < single particle, full contact ≈ multiple particles, partial contact < multiple particles, full contact, with values (mean ± standard deviation) of 0.20 ± 0.07, 0.38 ± 0.19, 0.37 ± 0.2, and 0.79 ± 0.18 V, respectively. Note that although there are relatively few single particle measurements herein, the Δ*E*_p_ values are consistent with our previous study (as shown in Figure 4b).^14^ As noted above, the SECCM probe size (i.e., 0.5 [from our previous study],^14^ 2 and 5 μm diameters) appears to have minimal influence on the measured Δ*E*_p_ values.

Comparing contact modes in Figure 4a, it is clear that the nature of the particle–support contact (i.e., *dry* vs *wet*) has a strong bearing on the measured Δ*E*_p_ values. Evidently, the dry contact modes give rise to apparently more facile Li^+^ (de)intercalation kinetics than the equivalent wet contact modes. Another important observation is that there is significantly more spread in the multiple particle data compared to the equivalent single particle data, with standard deviations of 0.19 V for multiple particle and 0.07 V for single particle measurements, respectively (both with partial contact). Plotting the logarithm of positive peak current, *i*_pa_ (a reasonable indicator of the number of particles probed) vs Δ*E*_p_ reveals a roughly linear relationship, as shown in Figure 4b and c. This relationship between current and peak separation could be due to kinetics, but also an element of resistive behavior, which we explore below through detailed simulations. We note that for this analysis, a larger total population of particles was probed in the multiple particle mode compared to single particles measurements (although, as mentioned, these are generally representative of our previous measurements).^14^

### Li^+^ (De)intercalation at ca. 100 LiMn_2_O_4_ Particles

**Figure 5:**
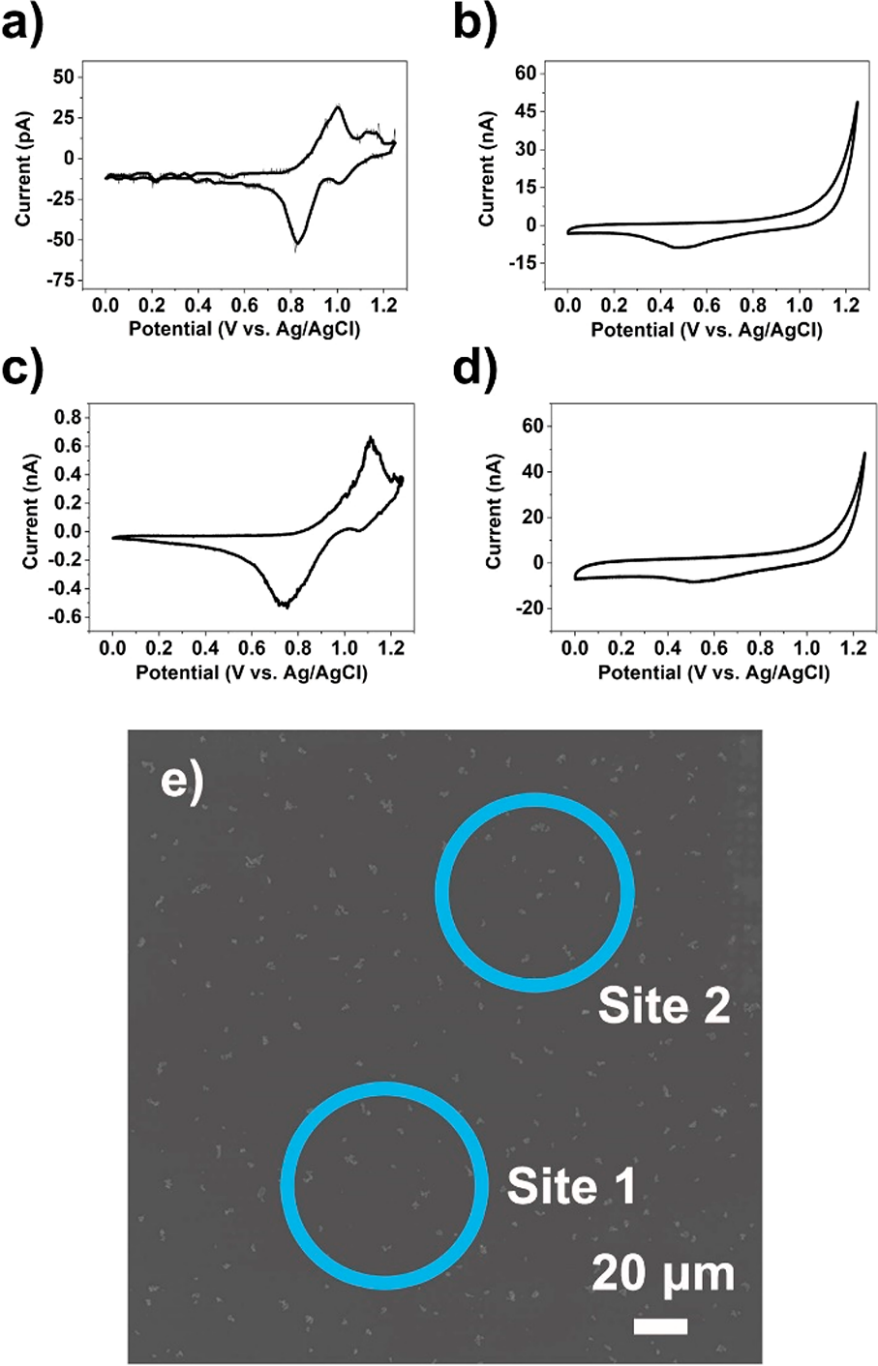
CV measurements from LiMn_2_O_4_ particles supported on GC, measured in *step-approach voltametric mode* at (a, c) the initial meniscus position (substrate dry) and (b, d) the final meniscus position (substrate wetted). Data in (a, b) and (c, d) were obtained from sites 1 and 2, respectively, as indicated in (e) the colocated SEM image. The CV measurements (ν = 1 V s^–1^) were obtained with probes of diameters ca. 70 μm filled with 1 M LiCl.

To simulate a “macroscopic” ensemble (much closer to a real electrode but without binders and conducting additives), a micropipette probe of diameter 70 μm (as shown in SI, Section 1, Figure S1c) was used to perform *step-approach cyclic voltammetric measurements*. As highlighted above, through precise positioning of the meniscus cell in 3D space, particle–support wetting status can be controlled, allowing the effects of particle resistance to be studied at the ensemble (ca. 100 particles) level. In contrast to the previous scanning experiments, the *step-approach voltammetric* measurements are carried out on a single point on the substrate. Figure 5 depicts the results of two such experiments. Figure 5a and c are examples of CVs following initial contact, creating a lower density ensemble with a *dry* particle–support contact. Both areas exhibit relatively facile Li^+^ (de)intercalation responses at LiMn_2_O_4_, with Δ*E*_p_ values of 0.17 and 0.36 V, respectively, which are at the lower end of the values found in the *voltammetric hopping mode* measurements in Figure 4a. Note that from the magnitude of the currents, it is evident that lower densities of LiMn_2_O_4_ particles were contacted in Figure 5a (*i*_pa_ ≈ 30 pA) compared to Figure 5c (*i*_pa_ ≈ 700 pA). This lower current, along with the smaller Δ*E*_p_, is also consistent with some degree of ohmic resistance in the measurement.

Figure 5b and d show examples where the probe is approached sufficiently close to the surface that the meniscus wets the GC support surface (as confirmed by SEM images of the area afterward, Figure S10b), simultaneously encapsulating ca. 15 LiMn_2_O_4_ agglomerates or 100 individual particles (shown in Figure 5e). In both cases, Figure 5b and d, the positive peak associated with Li^+^ deintercalation at LiMn_2_O_4_ completely overlaps with the background current from the GC support at >1 V, while the broad negative peak associated with Li^+^ intercalation shifts markedly in the negative direction, occurring at ca. 0.5 V on the return sweep. As in the previous experiments with smaller numbers of particles, the higher density of LiMn_2_O_4_ particles and/or *wet* particle–support contact significantly modifies the voltammetry.

**Figure 6:**
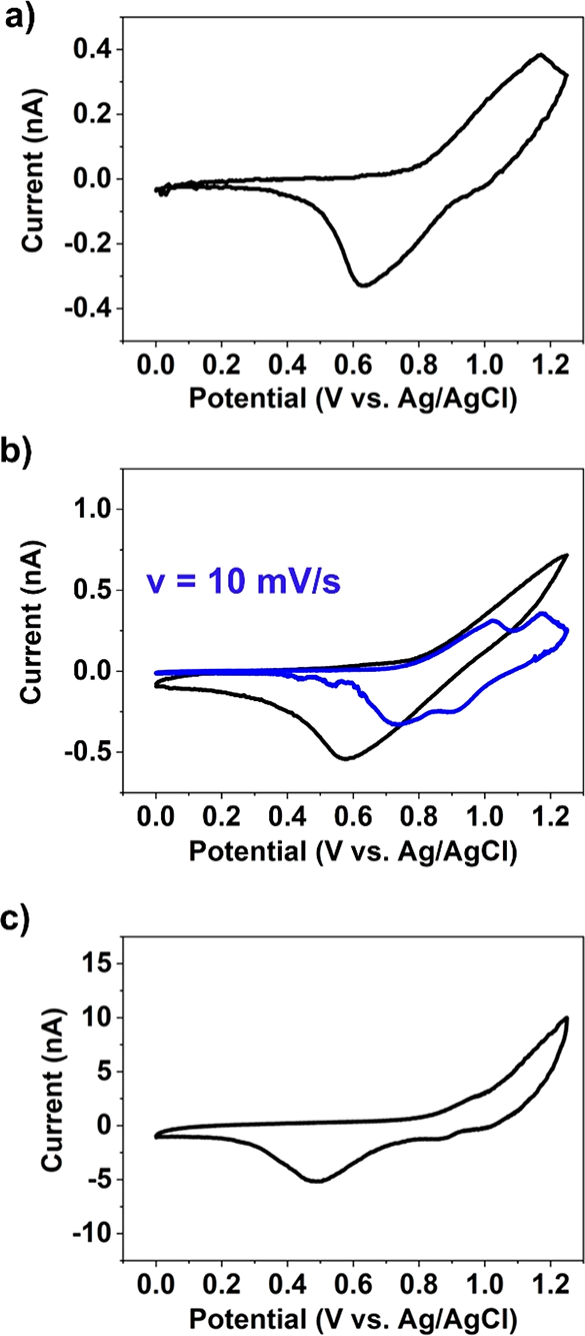
CV measurements from LiMn_2_O_4_ particles supported on HOPG, performed at increasing closer approach distance to give (a) low, (b) intermediate, and (c) high particle densities, with the CV in (c) also contacting the HOPG. The blue curve in (b) depicts a CV at the slower scan rate of 10 mV s^–1^. All other CV measurements (ν = 1 V s^–1^) were obtained with probes of diameter ca. 70 μm filled with 1 M LiCl.

Due to its relatively hydrophilic nature, only two wetting stages could be observed when GC was used as the support. To deconvolute the effects of interparticle interactions and *wet* contact resistance, it is necessary to study the intermediate wetting stages, i.e., where the LiMn_2_O_4_ particles are gradually encapsulated from top to bottom as the probe is lowered incrementally. Thus, an identical set of experiments was carried out using highly oriented pyrolytic graphite (HOPG) as the support, as shown in Figure 6. After exposure to the ambient atmosphere for extended periods (including for the preparation of the sample), HOPG is more hydrophobic and electrochemically inert than GC within the investigated potential range, as shown in the SI, Section 1, Figure S11.

A representative CV obtained upon initial contact with the HOPG-supported LiMn_2_O_4_ particles is shown in Figure 6a. The wave shape of the CV is comparable to that measured on GC for a similar current magnitude (i.e., *i*_pa_ ≈ 400 pA, comparable to Figure 5c), with a single pair of redox peaks located at 1.16/0.64 V. During this initial measurement, only the upper portions of the largest LiMn_2_O_4_ particles are contacted by the meniscus. As shown in Figure 6b, lowering the *z* position of the micropipette probe toward the surface led to an increase in the magnitude of the measured current due to a higher density of particles being contacted. Note that additional “intermediate” steps in this set of experiments (i.e., between initial contact with LiMn_2_O_4_ particles and ultimately wetting of the HOPG support) are presented in SI, Section 1, Figure S12. Comparing Figure 6a to b, upon contacting additional LiMn_2_O_4_ particles, Δ*E*_p_ increased from 0.52 to 0.67 V (assuming the switch potential of 1.25 V as the deintercalation potential in Figure 6b, although the reaction was not complete). Interestingly, also shown in Figure 6b, by decreasing the voltammetric sweep rate from 1 to 0.01 V s^–1^, the same set of particles exhibit the archetypal two-stage Li^+^ (de)intercalation response expected from bulk LiMn_2_O_4_ electrodes (see SI, Section S1, Figure S13).

Returning to the experiments described in Figure 6, upon further lowering the *z* position of the micropipette probe, the meniscus cell eventually breaks on the support surface, and the resulting CV is the sum of the LiMn_2_O_4_ particle responses and the HOPG signals, as shown in Figure 6c. Again, encapsulating additional LiMn_2_O_4_ particles and/or wetting the support (as evidenced by the meniscus deposit shown in Figure S14b), further hinders the apparent Li^+^ (de)intercalation kinetics, with no discernible positive peak and a significantly shifted negative peak (from ca. 0.58 to 0.49 V) when comparing Figure 6b to c. By comparing the CVs obtained from LiMn_2_O_4_/GC (Figure 5b or Figure 5d) and LiMn_2_O_4_/HOPG (Figure 6c), it seems that the nature of the support does not strongly influence *wet* contact resistance, as the morphologies of these curves are very similar, especially the characteristic broad peak occurring at the same position (ca. 0.5 V) during the negative scan.

After measurements across a range of length scales, several observations are clear: measuring multiple particles together gives rise to significantly shifted peaks (and increased Δ*E*_p_) in the CV; contacting the substrate in addition to the particles also causes significant shifts in the peaks; and particle size is not correlated with peak shift, but ensemble size is. As there can be multiple contributions to peak shifts in cyclic voltammetry, we turned to simulation to understand the observed trends.

### Finite Element Method Modeling of Single Particle Voltammetry

We carried out numerical simulation of the intercalation current–voltage response expected for single particles under a range of substrate and electrolyte contact geometries, starting from a model previously applied to single particle measurements.^37^ Details of the model can be found in the SI, Section S2. The model considers the coupled diffusion of Li^+^ with the intercalation flux on the particle boundary following Butler–Volmer kinetics, with rate constant, *k*, and an overpotential that varies with the local state of charge (SOC, SI, Figure S15). This model has successfully been applied to the case of single particle voltammetry at slow scan rates, where it was cast in 1D, and neglected the effect of the substrate electrode and solution, and the finite conductivity of the particle.^39^

More recent modeling has examined the interplay between surface kinetics and finite diffusion,^40^ as well as the role of particle shape,^49^ on single particle voltammetry, in both cases utilizing an analytical (Frumkin-type) approximation for the open circuit potential (OCP)–SOC relationship. However, to reproduce the voltammetry observed at high scan rates, it proved necessary to employ a more complex formulation of the OCP–SOC relationship fitted to experimental data.^41^ Here, we are interested in the effect on the voltammetry of variable particle–substrate contact and variable particle–solution contact (wetting). For computational efficiency, we use a 2D axis-symmetric geometry, with a particle of radius, *r*_0_, making contact with an area π*r*_c_^2^ of the substrate (SI, Figure S17). This approach has been applied previously to understand stress and heat generation in single LiMn_2_O_4_ particles,^50^ as well as to model voltammetry of single LiFePO_4_^51^ and LiNi_0.33_Mn_0.33_Co_0.33_O_2_^18^ particles.

**Figure 7:**
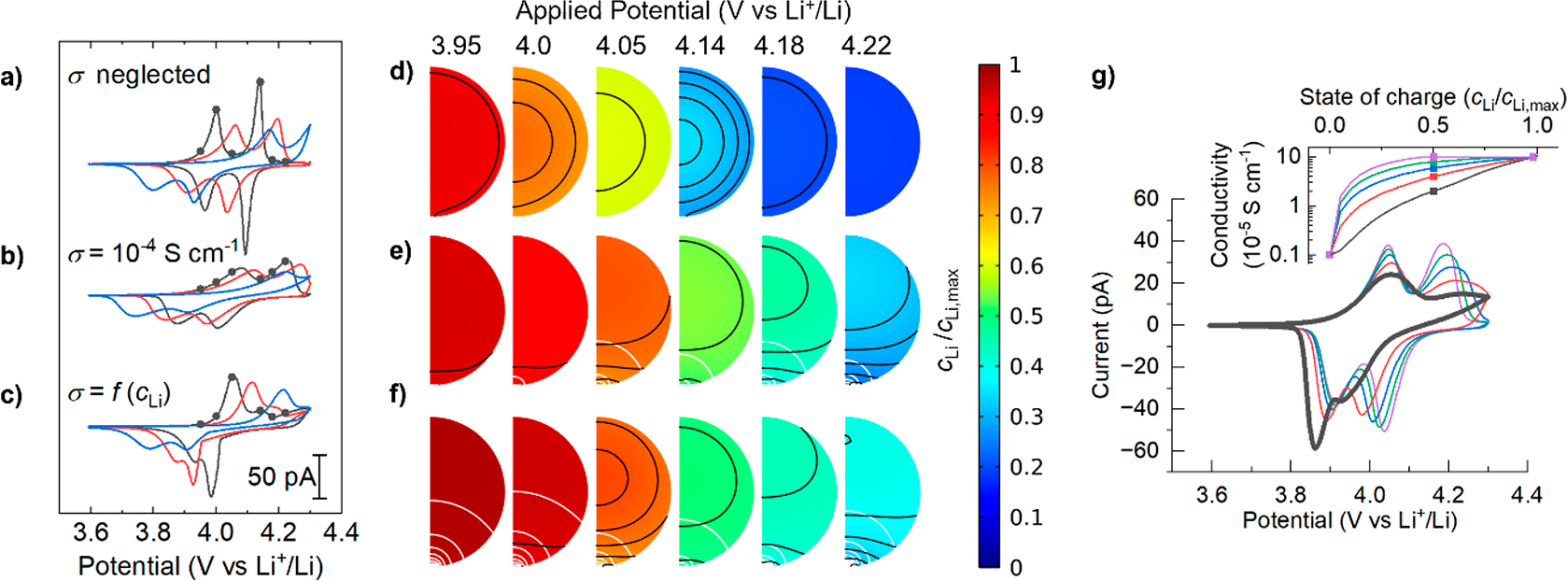
Simulated voltammetry of a single LiMn_2_O_4_ particle at 1 V s^–1^ with rate constants, *k* = 10^–3^ cm^5/2^ s^–1^ mol^–1/2^ (gray lines), *k* = 10^–4^ cm^5/2^ s^–1^ mol^–1/2^ (red lines) and *k* = 10^–5^ cm^5/2^ s^–1^ mol^–1/2^ (blue lines), for the cases of (a) neglected particle conductivity (σ neglected), (b) constant conductivity (σ = 10^–4^ S cm^–1^), and (c) variable conductivity (based on interpolated reported conductivities,^6^ σ = *f*(*c*_Li_)). *D*_Li_ = 2.2 × 10^–9^ cm^2^ s^–1^, *r*_0_ = 100 nm, *r*_c_ = 10 nm, ν = 1 V s^–1^. Concentration profiles at the marked potentials for the three cases with *k* = 10^–3^ mol^5/2^ mol^–1/2^ s^–1^ are shown for (d) neglected conductivity, (e) constant conductivity, and (f) variable conductivity. *c*_Li_/*c*_Li,max_ is the Li^+^ concentration normalized to the maximum concentration. Black contours represent 2% changes in normalized concentration. White contours show 10 mV changes in potential. (g) The transition from two equal to one dominant couple in voltammetry as an arbitrary SOC–conductivity profile (inset) is varied from an exponential shape (black) to a logarithmic shape (purple). *k* = 10^–3^ cm^5/2^ s^–1^ mol^–1/2^, ν = 1 V s^–1^.

Initially a 200 nm diameter particle was considered, with a solid-state Li^+^ diffusion coefficient, *D*_Li_ = 2.2 × 10^–9^ cm^2^ s^–1^,^39^ and rate constant, *k*, that was varied over a wide range to determine its effect on the shape of voltammetry (Figure 7). Note that the exchange current density is formally dependent on both the reduced (intercalated) Li^+^, the oxidized (solution) Li^+^, *and* the surface vacancy concentrations, such that *k* has units of cm^5/2^ s^–1^ mol^–1/2^. If the concentration of Li^+^(aq) at the surface (*c*_l_*) is not perturbed during intercalation (*vide infra*), then multiplication of *k* by (*c*_l_*)^(1-β)^ = 3.2 × 10^–2^ mol^1/2^ cm^–3/2^ (i.e., for *c*_l_* = 0.001 mol cm^–3^ and β = 0.5) recovers the standard rate constant *k*^0^ in units of cm s^–1^. At the magnitude previously used to fit data at slow scan rates (*k* = 10^–4^ cm^5/2^ s^–1^ mol^–1/2^),^39^ the peak separation of the most negative couple simulated (Δ*E*_p_^sim^) is of the same order of magnitude to that observed in the present experiments (Δ*E*_p_^sim^ = 151 mV, Δ*E*_p_^expt^ = 200 mV). The shape of the simulated voltammogram deviates significantly from the experiment, however, with a more symmetric, sharper pair of couples of similar magnitudes observed in the simulation, rather than a large, broad, asymmetric lower potential couple and a smaller higher potential couple seen in the experiments. Increasing *k* by an order of magnitude does not change the peak shapes, and only decreases the peak separation slightly, while decreasing *k* by an order of magnitude dramatically broadens and shifts the peaks, resulting in only one peak in the positive sweep (Figure 7a). The concentration profile remains relatively flat throughout the sweep at all values of *k* (Figure 7d), indicating predominantly kinetic control over the intercalation rates. Interestingly, varying *D*_Li_ to the maximum reported (10^–6^ cm^2^ s^–1^)^16^ only had a minor effect on the voltammetry, further supporting the notion of kinetic control. There was no significant deviation of the solution Li^+^ concentration from the bulk value under any of the conditions (and so is omitted in figures for clarity), consistent with the much slower diffusion of Li^+^ in the solid phase. We therefore re-examined the assumptions behind the original model^39^ to see what additional factors could be controlling the voltammetry.

Previously, it was assumed that the electrical conductivity of the spinel (upper estimate 10^–4^ S cm^–1^)^52^ was large enough to neglect the variation in potential throughout the particle, and therefore that the potential at the surface of the particle is the same as that applied to the substrate.^39^ For example, it was previously reported that for a current density of 0.6 mA cm^–2^, the potential drop across a distance of 10 μm would only be 5 mV. This cursory analysis assumes equal contact areas and a constant cross section, which is not the case for spherical particles. In addition to the particle resistance, the (Holm) contact resistance must also be considered, which is inversely proportional to the contact area.^53^ We therefore incorporated the electronic current into our model, via Ohm’s law, to allow simulation of a contact resistance between the substrate and the particle, and the potential drop across the particle itself. Initially, we used a value of conductivity, σ = 10^–6^ S cm^–1^, at the lower end of estimates,^6^ along with a 10 nm contact radius. This gave very resistive currents unlike anything observed experimentally. We therefore moved to the upper range of values, σ = 10^–4^ S cm^–1^.^6^ This led to a maximum potential drop across the particle of 38 mV (for *k* = 10^–3^ cm^5/2^ s^–1^ mol^–1/2^), resulting in the surface potential lagging the applied potential, broadening the voltammetric response (Figures 7b). The separation between the first positive and negative peaks increased significantly, with rate constant *k* = 10^–3^ cm^5/2^ s^–1^ mol^–1/2^ now giving the closest separation (Δ*E*_p_^sim^ = 205 mV) to that observed experimentally (Δ*E*_p_^expt^ = 200 mV). Clearly the electronic conductivity of the particles plays a fundamental role in voltammetry under these conditions and should not be neglected. It is important to consider the possible contribution of migration to the mass transport, given the electric fields present in the particle. It is clear from the small concentration gradients shown in Figure 7 that the current is primarily controlled by interfacial kinetics and not mass transport, and therefore that neglecting migration (which greatly simplifies the modeling) will not fundamentally change the interpretation of results.

The electronic conductivity of LiMn_2_O_4_ is known to be strongly dependent on the SOC, generally increasing with *x* in Li_*x*_Mn_2_O_4_ until *x* approaches 1.^6^ Next, we therefore included this factor in the model by making the conductivity dependent on the local Li^+^ concentration based on an (arbitrary piecewise cubic) interpolation of experimental SOC–conductivity data (see Figure S16 for the functional form).^6^ This dramatically modifies the voltammetric waveshape, with a large, broad low potential peak followed by a smaller high potential peak on the positive sweep, and conversely, a large, broad high potential peak and a smaller low potential peak on the negative sweep (Figure 7c). Qualitatively, the distinct changes in waveshape and the relative height of the two peaks in the positive sweep agree with the experimental voltammetry, although a difference remains in the negative sweep. To investigate the role of the conductivity function further, we examined a range of arbitrary ramps in conductivity with SOC from 10^–6^ S cm^–1^ at low SOC to 10^–4^ S cm^–1^ at high SOC, showing logarithmic-type to exponential-type increases (inset, Figure 7g). For the logarithmic-type increase, where the conductivity remains high for a large window of SOC, the CV resembles the constant conductivity case, with two pairs of peaks of equal magnitude. As the conductivity function bows downward, to resemble an exponential-type increase, the second oxidation peak deceases in magnitude and broadens, while the corresponding reduction peak slightly decreases in magnitude and shifts to more negative potential. This results in a voltammogram which much more closely resembles that observed experimentally, with a smaller second pair of peaks at high potential, and a general trend to reduction/oxidation peak ratios greater than 1. We note that a monotonic increase in conductivity with SOC is an oversimplification (e.g., conductivity decreases at the highest SOC^6^); however, in the absence of an accepted high resolution experimental data set, it is sufficient to show the significant effect of the conductivity–SOC relationship on voltammetry (and hence rapid charge/discharge). Significantly, this important information is all but lost at the slow scan rates typically used in voltammetry of battery particles (Figure S18), highlighting the advantage of using SECCM to study fast charging materials.

**Figure 8:**
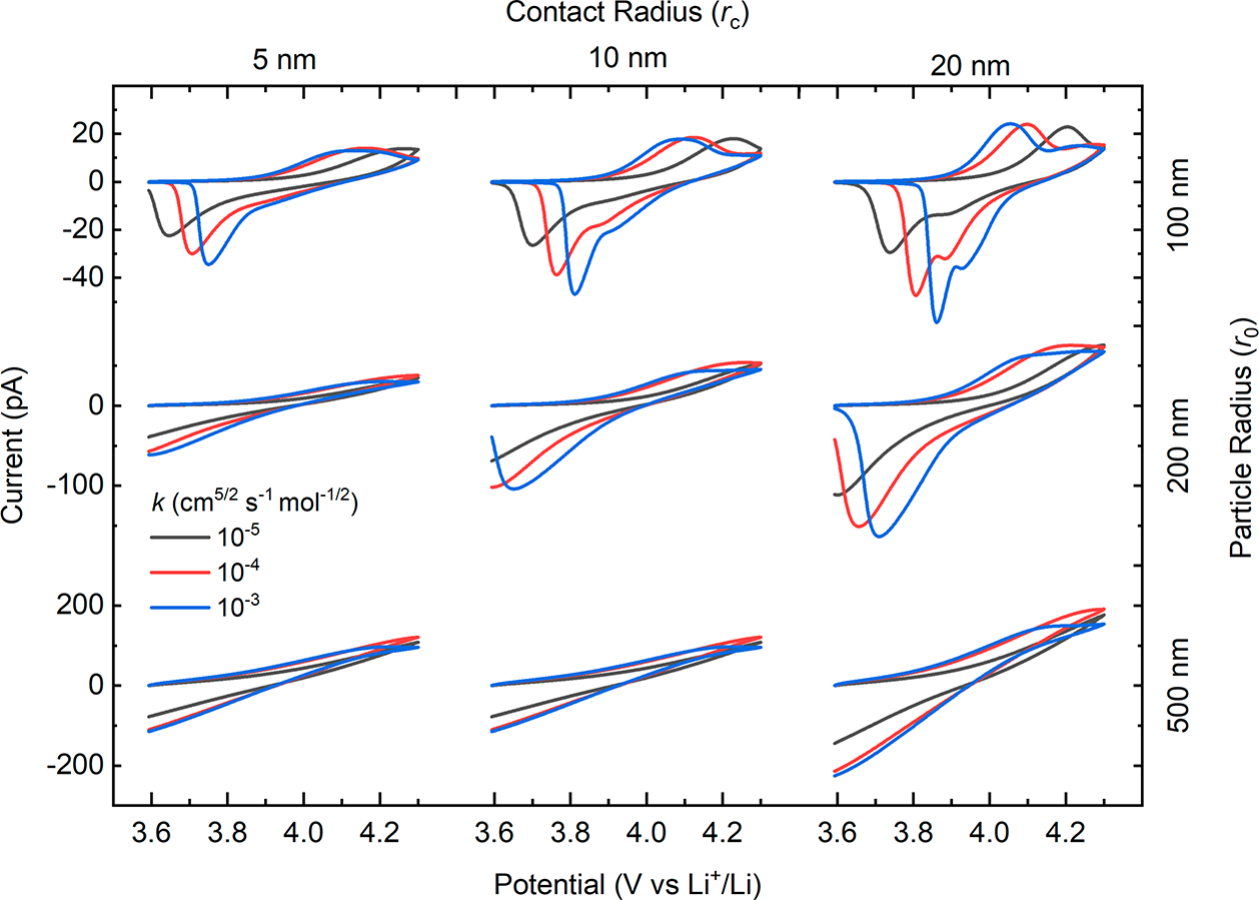
Effect of particle size and contact area on voltammogram shape. ν = 1 V s^–1^. *D*_Li_ = 2.2 × 10^–9^ cm^2^ s^–1^. An arbitrary exponential-type SOC–conductivity curve was used (black curve, Figure 7g, inset).

The dramatic change in wave shape when considering variable SOC–conductivity suggests that particle size and contact area will be particularly important. We therefore examined the simulated voltammetric response for a range radii of both particle and contact with *k* (cm^5/2^ s^–1^ mol^–1/2^) = 10^–5^, 10^–4^, and 10^–3^ and the exponential-type conductivity function (black curve, Figure 7g, inset) used above and plot the results in Figure 8.

For the smallest particle size examined (*r*_0_ = 100 nm) with the largest contact area (*r*_c_ = 20 nm) and the fastest rate constant (*k* = 10^–3^ cm^5/2^ s^–1^ mol^–1/2^), the voltammogram qualitatively resembles those obtained experimentally (e.g., Figure 2a) and shows similar peak separation (Table 1). However, as the contact area decreases, there is a subtle loss of definition in the peaks, along with a dramatic change in peak separation, indicative of the increasing importance of the ohmic term. The effect is much more dramatic at larger particle sizes, where the current is higher, due to the larger surface area, and the current path through the material is longer, both factors contributing to much greater voltage drop from the substrate/particle contact to the particle/solution boundary. Interestingly, the effect of the rate constant value is most obvious in the negative peak, which decreases in magnitude and shifts to more negative potentials with decreasing *k*. As expected, resistive effects diminish at slower scan rates where the current is lower (Figure S19), although some peak splitting is observed in the larger particles, being most significant for the largest particle with the smallest contact area (*r*_0_ = 500 nm, *r*_c_ = 5 nm). Again, these results highlight the importance of fast scan rate SECCM in revealing aspects of material performance (i.e., morphology dependence) that would be lost with slower measurements.

**Table 1:** Peak Separations for Most Negative Potential Couple in Figure 8 for *r*_0_ = 100 nm

	Δ*E*_p_^sim^ (mV)		
	*k* (cm^5/2^ s^–1^ mol^–1/2^)		
**r*_c_ (nm)*	10^–5^	10^–4^	10^–3^
5	618	454	380
10	529	360	280
20	466	293	192

**Figure 9:**
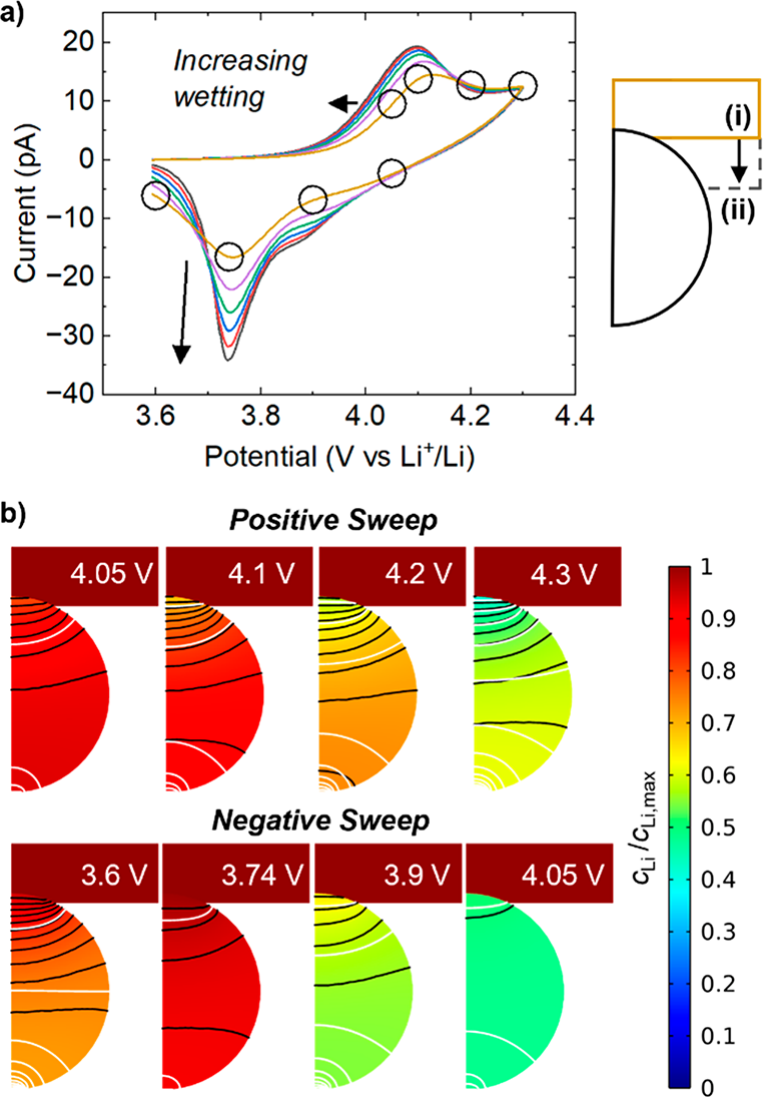
Effect of wetted area on voltammogram shape. (a) Cyclic voltammograms at decreasing meniscus heights, starting from position (i) and ending at position (ii), i.e., changed by 10 nm between each step. *k* = 10^–3^ cm^5/2^ s^–1^ mol^–1/2^, ν = 1 V s^–1^, *r*_0_ = 100 nm, *r*_c_ = 10 nm, *D*_Li_ = 2.2 × 10^–9^ cm^2^ s^–1^. The same arbitrary exponential-type SOC–conductivity curve as Figure 8 was used. (b) Concentration profiles at the position indicated by circles in (a). *c*_Li_/*c*_Li,max_ is the Li^+^ concentration normalized to the maximum concentration. Black contours represent 2% changes in normalized concentration. White contours show 10 mV changes in potential.

The effect of contacting different amounts of particle surface by the droplet (i.e., different degrees of particle wetting) can also be simulated with this model, by varying the height of the solution domain relative to that of the particle (Figure 9; see simulation geometry in SI, Figure S20). As noted above, the change in wetting is most noticeable in the negative peak, which increases in magnitude, but remains at the same potential as the wetting increases (Figure 9a). The positive peak increases and shifts slightly to less positive values with increased wetting, indicating that the increasing apparent resistance in the *step-approach voltammetry* experiments (Figure 6) arises from the dominant contribution of the large number of particles that have newly made contact with the meniscus in this step and only have low wetted areas (and hence high resistance, *vide infra*), rather than any intrinsic change in kinetics/voltammetry seen when the contact with individual particles is increased. This interpretation is also consistent with the appearance of small shoulder peaks showing smaller Δ*E*_p_ values in the fully wetted CV (Figure 6c), where initially poorly wetted particles will have fully wetted, increasing their ionic contact areas and decreasing their peak separations as a result.

**Figure 10:**
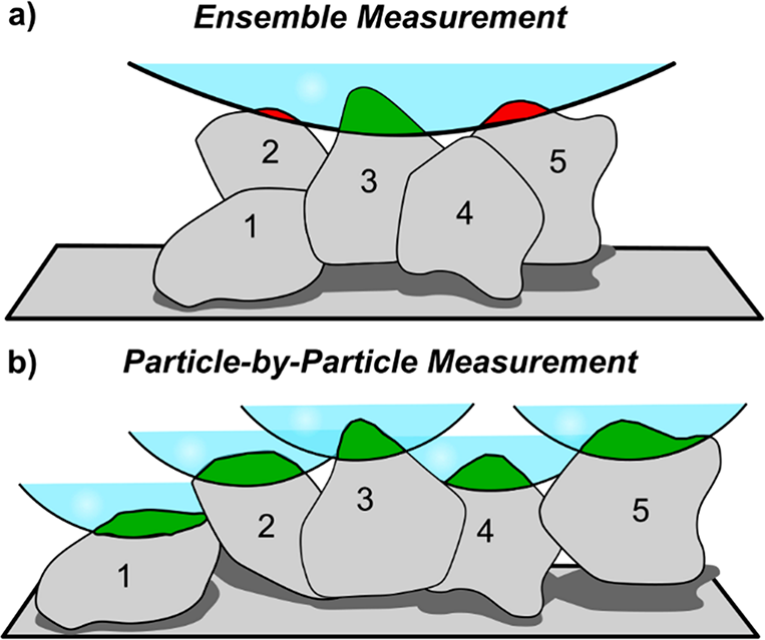
Two outcomes from measuring the same five particles with SECCM. (a) The particles are clustered so that the meniscus area covers all particles such that the tallest particle halts the approach, leading to variable wetting of the other particles. (b) The particles are spaced out so that the meniscus area only covers one particle at a time. Each particle is therefore able to halt the meniscus approach with a similar amount of wetting. The current threshold is reached when a given area is contacted (shown in green). Low wetted areas are shown in red.

The variable wetting behavior also serves to rationalize the difference between single and multiple particle experiments. In single particle (*dry* substrate contact) measurements, good meniscus–particle contact is a necessary condition for the SECCM probe approach, as pipet translation is automatically halted only when a (consistent) threshold current (i.e., wetted area) is detected. In contrast, in multiple particle experiments, it is only necessary for a single particle to be contacted by the meniscus before the approach is halted, resulting in a range of wetting conditions for the remaining particles encapsulated by the meniscus (Figure 10a). This range of wetted contact areas will result in a wider range of interparticle resistances and peak positions, which on average will appear more resistive/kinetically hindered than the single particle measurements. If meniscus contact was made with each particle individually, ensuring consistent wetting, it is likely their response when averaged would be faster, although differences in particle shape, size, and relative orientation would still lead to small differences in wetting, resistance, and therefore waveshape (Figure 10b).

The modeling above emphasizes the importance of contact resistance on single particle measurements, and this can be extended to the differences observed in measurements with *wet* and *dry* substrate contacts, where the contact resistance evidently increases when the substrate is wetted. When dry, the contact area is determined by the forces acting on the particle (mainly van der Waals interactions). On wetting the contact area, electrostatic interactions between the electrical double layers of the particle and the substrate become important. As the particle and substrate are at similar potentials (contact resistance notwithstanding), they are likely to have double layer charges of the same sign (although noting some dependence on the potential of zero charge of each material), which would contribute a repulsive force, counteracting some of the attractive force and decreasing the contact area. Potential-dependent double layer repulsion is seen in experiments with metallic particles settling on electrodes; indeed, this is the mechanism behind ‘contact charge electrophoresis’ in which particles are oscillated between electrodes by repetitive cycles of contact–charging–repulsion.^54^ Such effects appear to have received little attention in the battery field, although a previous report on primary alkaline MnO_2_ batteries noted that the electronic conductivity of the dry matrix decreased up to 30% on wetting with electrolyte, but not with deionized water.^55^ This is consistent with an electrical double layer repulsion model, where surface charging is significantly reduced in the absence of charge-compensating solution ions.

## Conclusions

SECCM allows high throughout, targeted electrochemistry of battery materials at fast scan rates in a range of environments, from isolated single particles on a conductive support to large numbers of aggregated clusters. Fine control over the height of the pipet meniscus further generates a range of wetting conditions, allowing the effects of electronic and ionic contacts with the active particle to be observed and decoupled in ways not possible at the macroscale. When ensembles of varying sizes are systematically probed using fast scan rate voltammetry, a pattern of increasing peak separation is observed that generally increases from single particles to ensembles and from partial wetting by the electrolyte (leaving the substrate unwetted) to full wetting including the substrate.

Simulations reveal that voltammetry at fast scan rates contains a significant contribution from the electronic resistance of the particles and their random orientation and contact with the substrate, in contrast to voltammetry at slow scan rates which is not as sensitive to such factors. Expanding the scan rate range, as possible through nanoscale and microscale measurements, as described herein, provides a means of revealing and testing these factors. Electrolyte contact also has some role in determining the waveshape, where the predominant effect is on the negative going sweep. The solid-state Li^+^ diffusion coefficient in the material, over a reasonable range of values, contributes least to the voltammetric response.

This work highlights that, in nonmetallic redox systems, material conductivity becomes an important consideration for small scale measurements like SECCM. The findings herein extrapolate to composite electrodes and suggest that optimizing electronic contact between particles should take precedent over electrolyte contact. Indeed, this is borne out by the continued success of carbon-coating active particles.^2,56^

## Supporting Information Available

STEM images of micropipettes used, cyclic voltammograms and SEM from each pixel analyzed, tables of associated peak positions, details of FEM modeling, plots of model input parameters, and simulated voltammograms at slow scan rates (PDF)
