# Supplementary material for: Multiscale Electrochemistry of Lithium Manganese Oxide (LiMn2O4): From Single Particles to Ensembles and Degrees of Electrolyte Wetting
Source: ACS Sustain Chem Eng. 2023 Jan 13;11(4):1459–71. doi: 10.1021/acssuschemeng.2c06075 (PMC9890564; doi:10.1021/acssuschemeng.2c06075)
Supplement: Supplementary file 1 — sc2c06075_si_001.pdf [file sc2c06075_si_001.pdf]

Supporting Information

Multiscale Electrochemistry of Lithium Manganese Oxide ($LiMn_2O_4$): From Single Particles to Ensembles and Degrees of Electrolyte Wetting

Binglin Tao,[1,2‡] Ian J. McPherson,[1,3‡] Enrico Daviddi,[1] Cameron L. Bentley[4]* and Patrick R. Unwin[1]*
[1]*Department of Chemistry, University of Warwick, Coventry CV4 7AL, U.K.*
[2]*Present Address: Talga Technologies Limited, Cambridge, CB24 9ZR, U.K.*
[3]*Present Address: Department of Chemistry, Loughborough University, Loughborough, LE11 3TU, U.K.*
[4]*School of Chemistry, Monash University, Clayton 3800 VIC, Australia*
[‡] These authors contributed equally to this work
Corresponding authors: cameron.bentley@monash.edu, p.r.unwin@warwick.ac.uk

Contents

1. Supporting Figures S2
2. Finite Element Method Modeling S16
3. References S22

Number of pages: 22
Number of figures: 20
Number of tables: 4

1. Supporting Figures

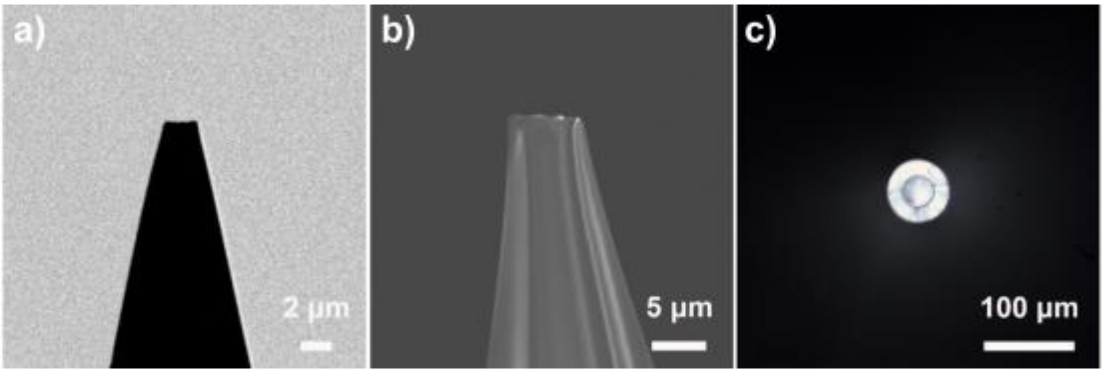

Figure S1. Micropipette with different sizes used in this work: **a)** STEM image of a typical nanopipet with diameter of 2 µm, **b)** SEM image of a nanopipet with diameter of 5 µm, **c)** optical image of a nanopipet with diameter of 70 µm.

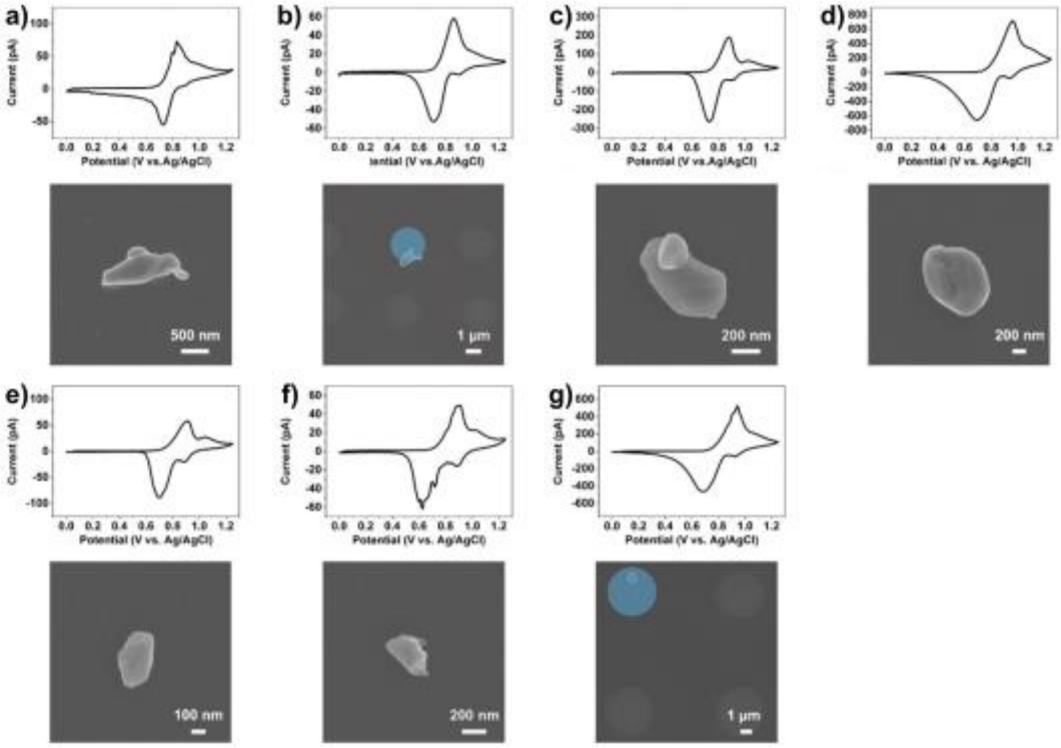

Figure S2. CVs and corresponding SEM images from $LiMn_2O_4$ particles supported on glassy carbon in the *single* particle, *partial* (*dry*) particle-support contact mode. **(a-f)** CV measurements (v = 1 V s^{-1}) were obtained with probes of diameter *ca.* 2 µm filled with 1 M LiCl solution. **g)** CV measurements v = 1 V s^{-1}) were obtained with probes of diameter *ca.* 5 µm filled with 1 M LiCl solution.

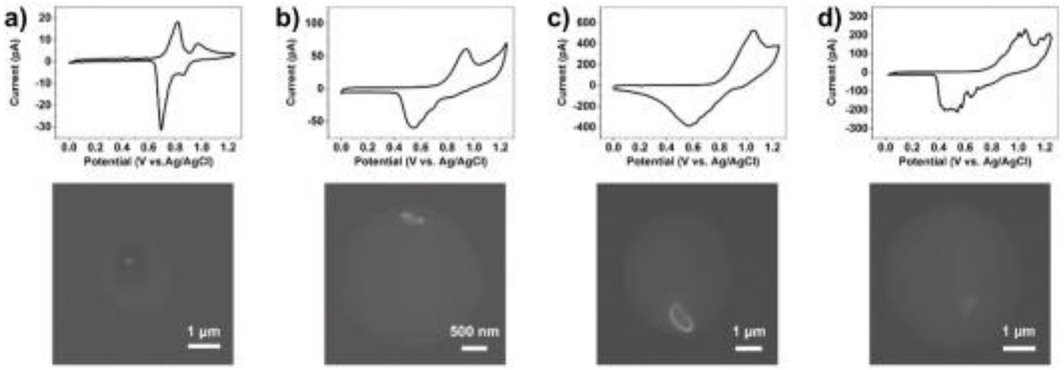

Figure S3. CVs and corresponding SEM images from $LiMn_2O_4$ particles supported on glassy carbon in the *single* particle *full* contact (*wet*) particle-support contact. **(a-b)** CV measurements (v = 1 V s^{-1}) were obtained with probes of diameter *ca.* 2 µm filled with 1 M LiCl solution. **(c-d)** CV measurements (v = 1 V s^{-1}) were obtained with probes of diameter *ca.* 5 µm filled with 1 M LiCl solution.

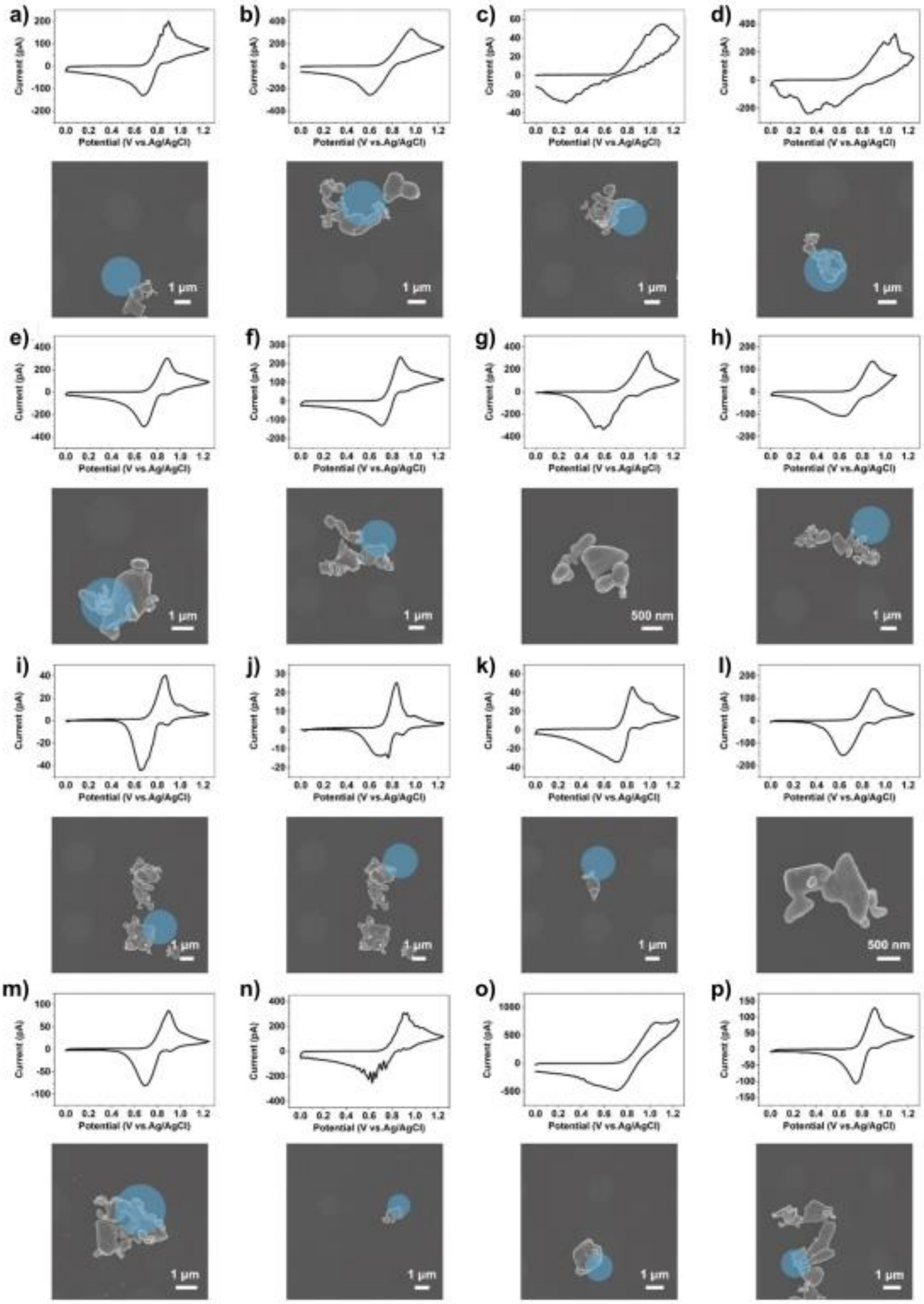

Figure S4. CVs and corresponding SEM images from $LiMn_2O_4$ particles supported on glassy carbon in the *multiple* particle, *partial* (*dry*) particle-support contact mode. **(a-p)** CV measurements (v = 1 V s^{-1}) were obtained with probes of diameter *ca.* 2 µm filled with 1 M LiCl solution. Blue circles indicate meniscus position.

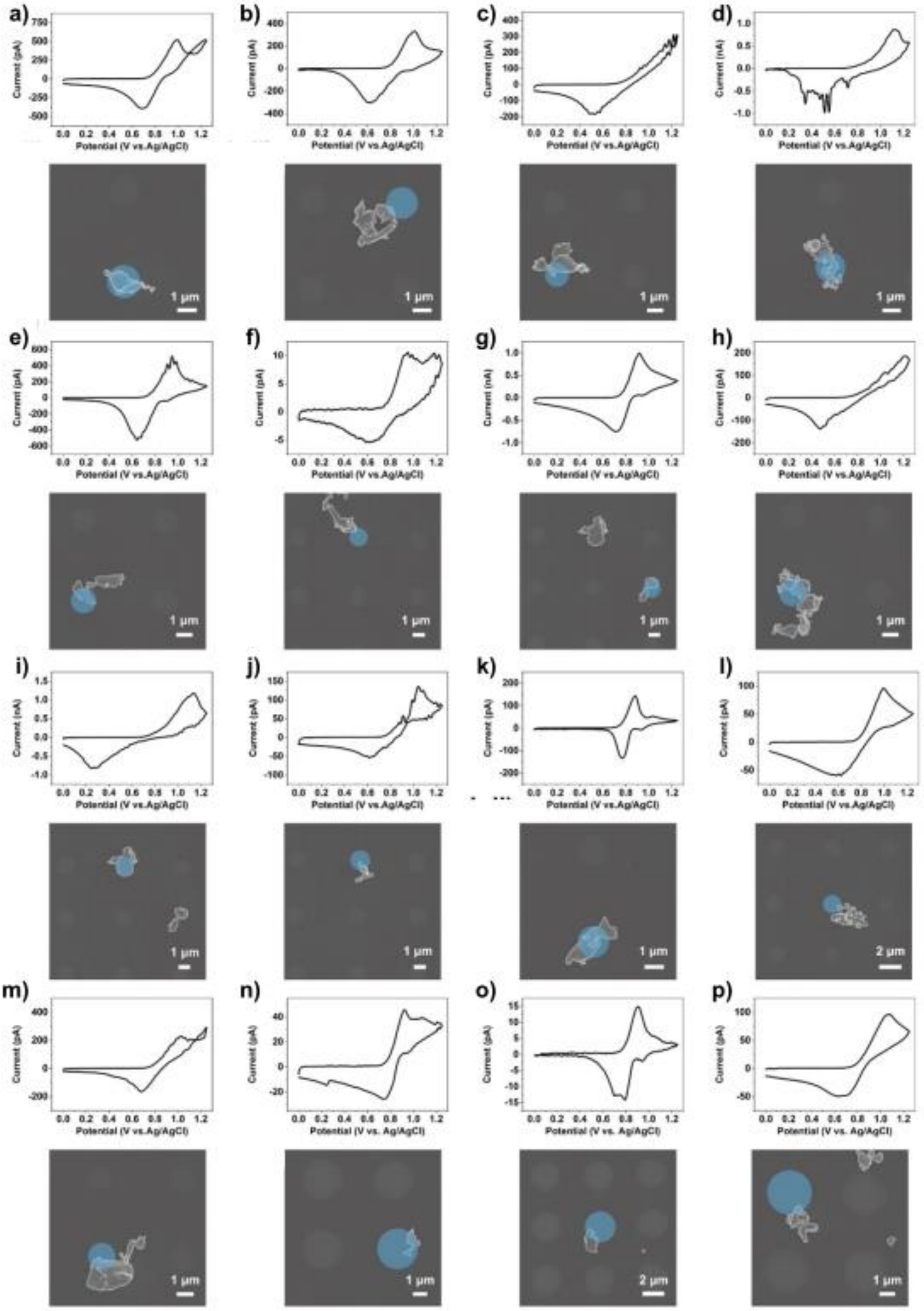

Figure S5. CVs and corresponding SEM images from $LiMn_2O_4$ particles supported on glassy carbon in the *multiple* particle, *partial* (*dry*) particle-support contact mode. **(a-p)** CV measurements (v = 1 V s^{-1}) were obtained with probes of diameter *ca.* 2 µm filled with 1 M LiCl solution. **Figure S5** is the continuation of **Figure S4**. Blue circles indicate meniscus position.

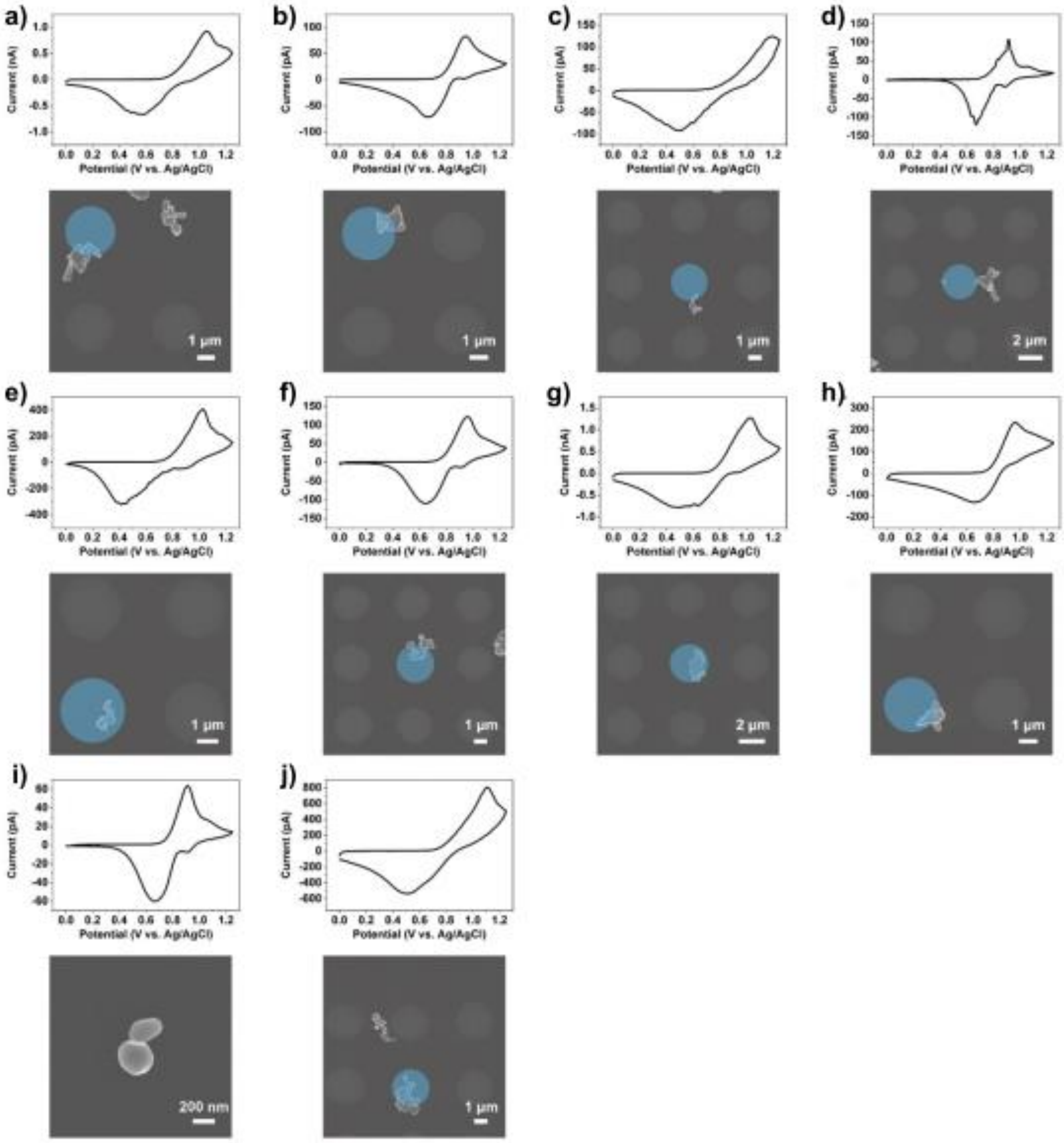

Figure S6. CVs and corresponding SEM images from $LiMn_2O_4$ particles supported on glassy carbon in the *multiple* particle, *partial* (*dry*) particle-support contact mode. **(a-j)** CV measurements (v = 1 V s^{-1}) were obtained with probes of diameter *ca.* 2 µm filled with 1 M LiCl solution. **Figure S6** is the continuation of **Figure S4 and Figure S5**. Blue circles indicate meniscus position.

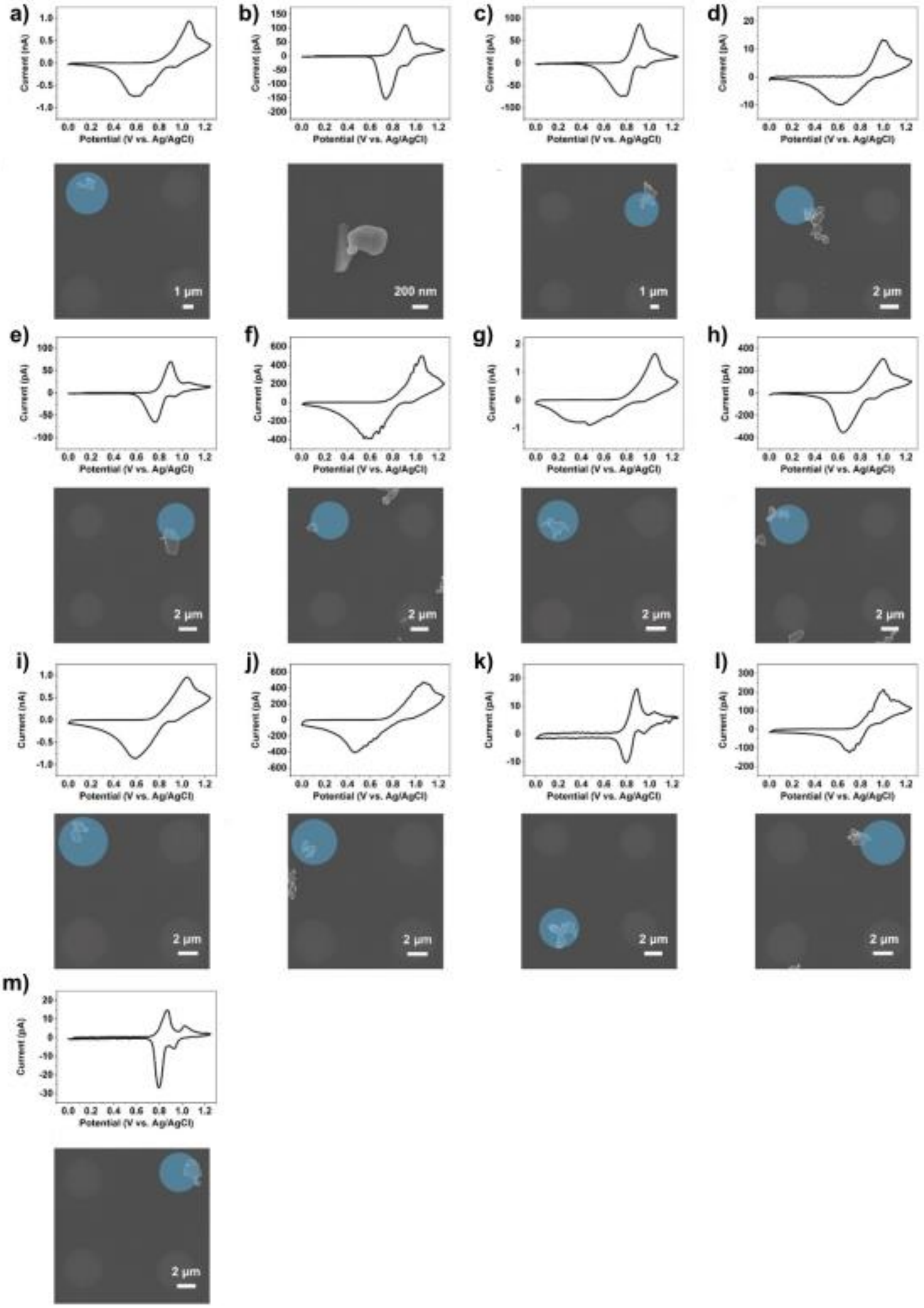

Figure S7. CVs and corresponding SEM images from $LiMn_2O_4$ particles supported on glassy carbon in the *multiple* particle *partial* (*dry)* particle-support contact mode. **(a-m)** CV measurements (v = 1 V s^{-1}) were obtained with probes of diameter *ca.* 5 µm filled with 1 M LiCl solution. Blue circles indicate meniscus position.

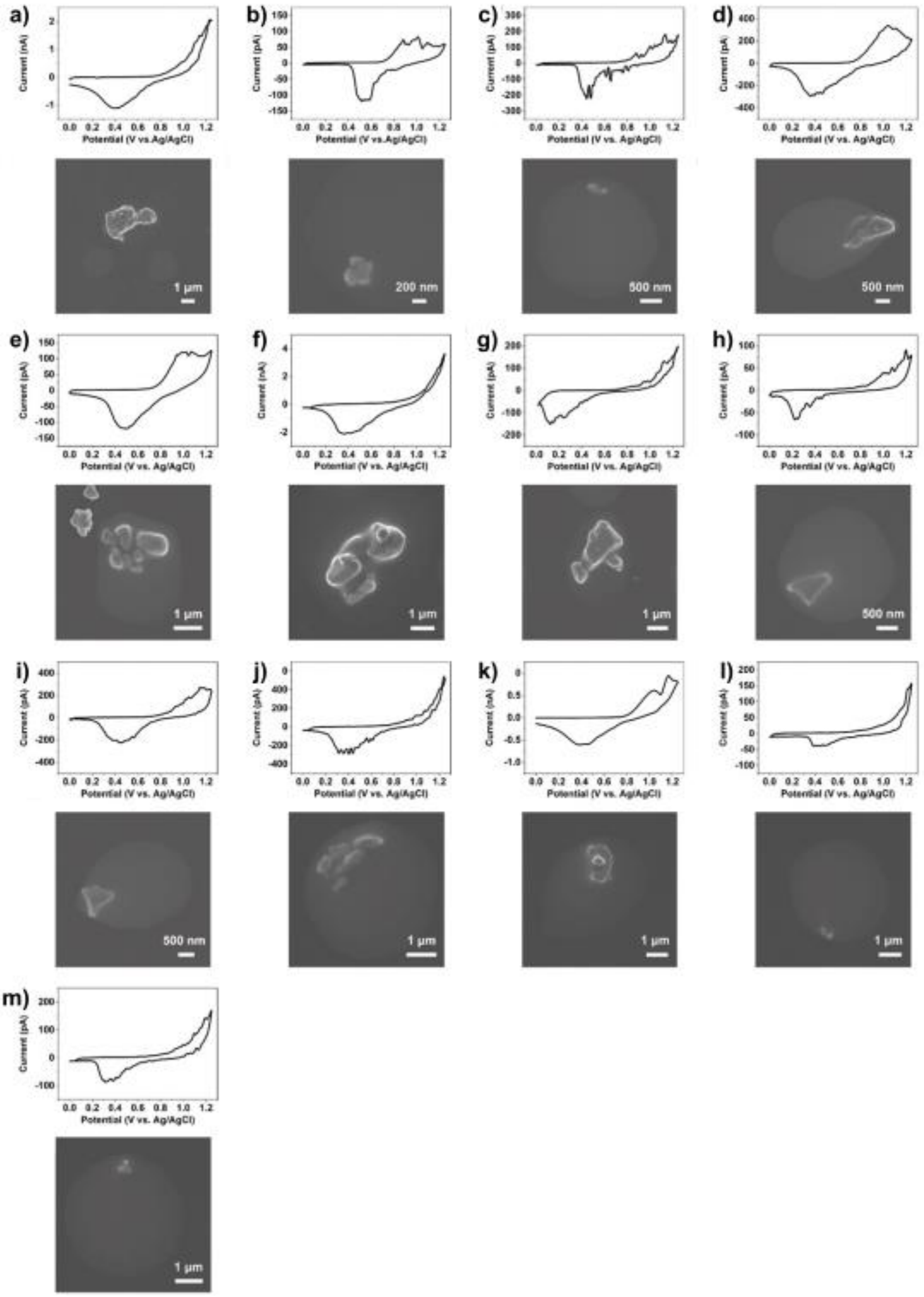

Figure S8. CVs and corresponding SEM images from $LiMn_2O_4$ particles supported on glassy carbon in the *multiple* particle, *full* (*wet)* particle-support contact mode. **(a-i)** CV measurements (v = 1 V s^{-1}) were obtained with probes of diameter *ca.* 2 µm filled with 1 M LiCl solution. **(j-m)** CV measurements (v = 1 V s^{-1}) were obtained with probes of diameter *ca.* 5 µm filled with 1 M LiCl solution. Blue circles

indicate meniscus position.

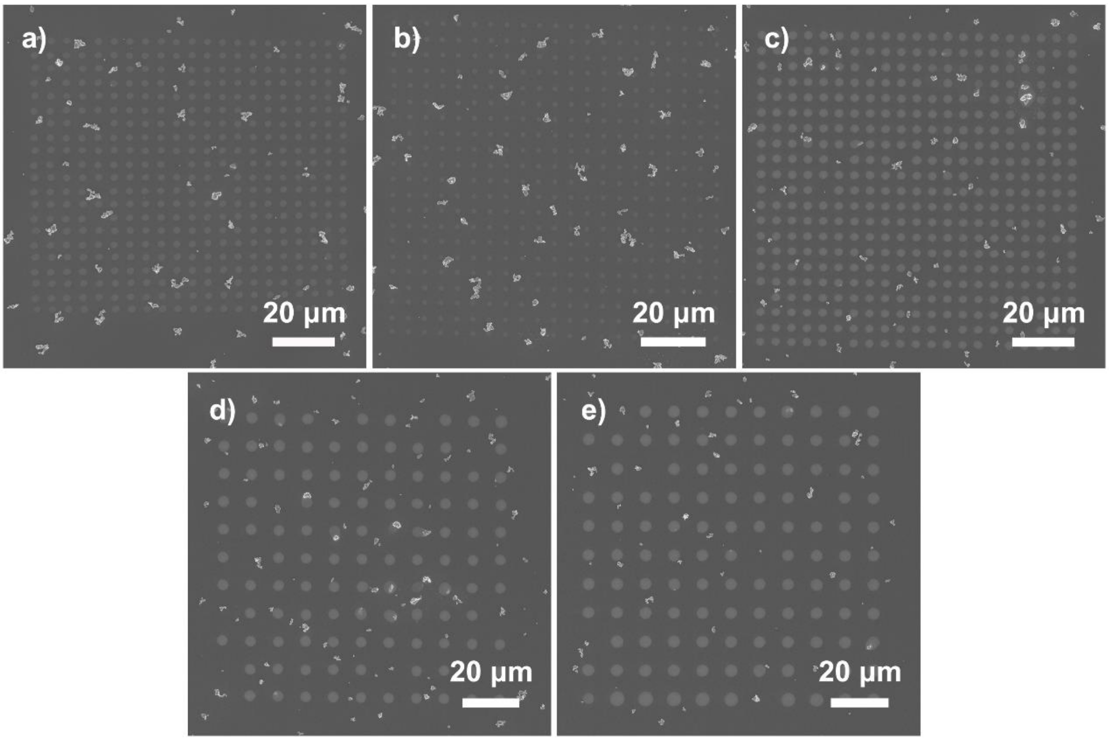

Figure S9. SEM images of the scanning areas of the cyclic voltammetry measurements of individual and aggregated $LiMn_2O_4$ particles supported on glassy carbon. **a)**, **b)** and **c)** performed with tips of diameter *ca.* 2 µm with hopping distance of 5 µm. **d)** and **e)** performed with tips of diameter *ca.* 5 µm with hopping distance of 10 µm.

Table S1. Electrochemical characteristics of four different electrolyte-particle(s)-glassy carbon contact modes, derived from 5 different cyclic voltammetry scans (the corresponding SEM images shown in **Figure S2**). The particle sequence in this table is the same with **Figure S3**-**S9**.

Tip size (μm)	Anodic Peak Voltage (V)	Anodic Peak Current (nA)	Cathodic Peak Voltage (V)	Cathodic Peak Current (nA)	Peak Separ ation (V)	Multiple or Single Particle(s)	Contact with Substrate or not
2	0.83	72	0.73	-55	0.1	S	N
2	0.86	58	0.71	-53	0.15	S	N
2	0.88	194	0.73	-263	0.15	S	N
2	0.96	722	0.7	-660	0.26	S	N
2	0.91	60	0.7	-90	0.21	S	N
2	0.9	50	0.63	-62	0.27	S	N
5	0.94	531	0.68	-468	0.26	S	N
2	0.81	18	0.7	-31	0.11	S	Y
2	0.94	62	0.54	-61	0.4	S	Y
5	1.03	531	0.58	-388	0.45	S	Y
5	1.05	233	0.49	-194	0.56	S	Y
2	0.89	187	0.68	-130	0.21	M	N
2	0.97	331	0.6	-256	0.37	M	N
2	1.1	55	0.25	-29	0.85	M	N
2	1.09	330	0.33	-237	0.76	M	N
2	0.89	300	0.68	-306	0.21	M	N
2	0.87	235	0.7	-129	0.17	M	N
2	0.97	354	0.55	-310	0.42	M	N
2	0.89	136	0.62	-108	0.27	M	N
2	0.87	40	0.65	-44	0.22	M	N
2	0.84	25	0.71	-14	0.13	M	N
2	0.85	46	0.71	-34	0.14	M	N
2	0.9	140	0.63	-154	0.27	M	N
2	0.9	85	0.7	-81	0.2	M	N
2	0.92	297	0.63	-243	0.29	M	N
2	1.05	729	0.71	-486	0.34	M	N
2	0.91	129	0.75	-106	0.16	M	N
2	1	520	0.7	-396	0.3	M	N
2	1	331	0.63	-303	0.37	M	N
2	1.25	311	0.52	-183	0.73	M	N
2	1.13	870	0.47	-660	0.66	M	N
2	0.95	517	0.64	-525	0.31	M	N
2	0.95	10.5	0.6	-5.4	0.35	M	N
2	0.92	1000	0.71	-754	0.21	M	N

2	1.22	188	0.47	-137	0.75	M	N
2	1.14	1180	0.25	-822	0.89	M	N
2	1.04	135	0.62	-54	0.42	M	N
2	0.88	145	0.7	-132	0.18	M	N
2	0.99	97	0.6	-58	0.39	M	N
2	1.02	230	0.68	-165	0.34	M	N
2	0.92	46	0.75	-26	0.17	M	N
2	0.9	15	0.8	-14	0.1	M	N
2	1.07	97	0.66	-49	0.41	M	N
2	1.05	933	0.54	-660	0.51	M	N
2	1.18	124	0.5	-93	0.68	M	N
2	0.94	84	0.66	-73	0.28	M	N
2	0.91	109	0.67	-120	0.24	M	N
2	1.03	413	0.42	-324	0.61	M	N
2	0.96	125	0.65	-110	0.31	M	N
2	1.03	1290	0.48	-789	0.55	M	N
2	0.96	238	0.67	-135	0.29	M	N
2	0.91	64	0.67	-60	0.24	M	N
2	1.1	810	0.51	-536	0.59	M	N
5	1.06	953	0.6	-741	0.46	M	N
5	0.91	115	0.74	-155	0.17	M	N
5	0.91	89	0.77	-74	0.14	M	N
5	1	13.3	0.61	-9.9	0.39	M	N
5	0.9	72	0.76	-66	0.14	M	N
5	1.05	502	0.6	-389	0.45	M	N
5	1.05	1692	0.47	-906	0.58	M	N
5	1	311	0.65	-355	0.35	M	N
5	1.05	974	0.59	-865	0.46	M	N
5	1.06	482	0.47	-412	0.59	M	N
5	0.89	16.5	0.8	-10.3	0.09	M	N
5	1	216	0.7	-127	0.3	M	N
5	0.87	15	0.8	-27	0.07	M	N
2	1.25	2050	0.4	-1107	0.85	M	Y
2	1.02	82	0.54	-114	0.48	M	Y
2	1.13	182	0.44	-220	0.69	M	Y
2	1.04	346	0.36	-293	0.68	M	Y
2	1.07	124	0.5	-120	0.57	M	Y
2	1.25	3650	0.37	-2125	0.88	M	Y
2	1.25	200	0.12	-152	1.13	M	Y
2	1.2	90	0.22	-65	0.98	M	Y
2	1.15	278	0.45	-230	0.7	M	Y
5	1.24	546	0.4	-292	0.84	M	Y
5	1.04	625	0.38	-611	0.66	M	Y

5	1.25	159	0.43	-38	0.82	M	Y
5	1.25	171	0.31	-88	0.94	M	Y

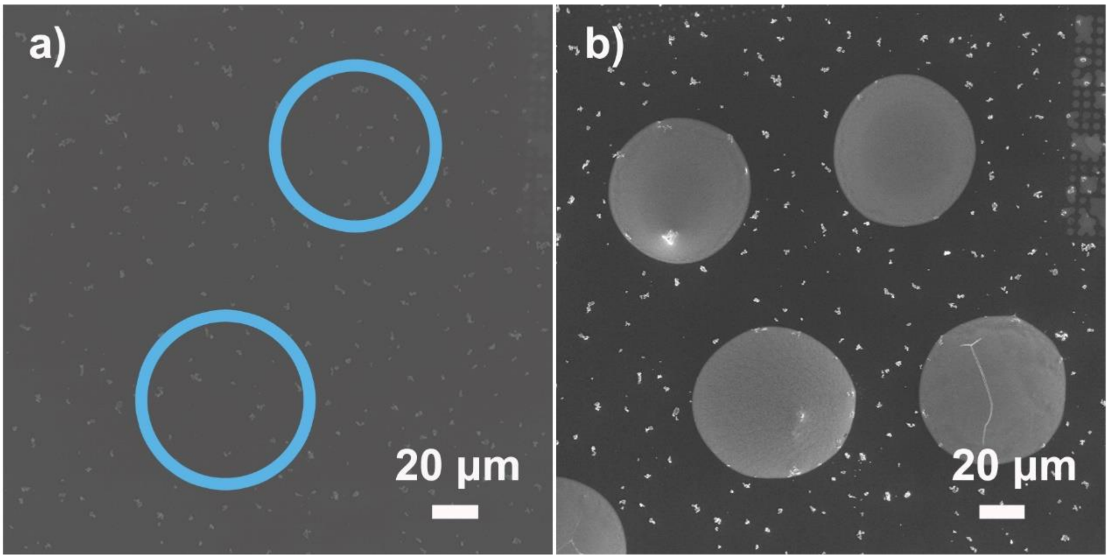

Figure S10. Cyclic voltammetric experiments performed on $LiMn_2O_4$ particles supported glassy carbon electrode with tips of diameter *ca.* 70 µm. Before **a)** and after **b)** experiment. The probed areas are indicated by blue circles in **a)**.

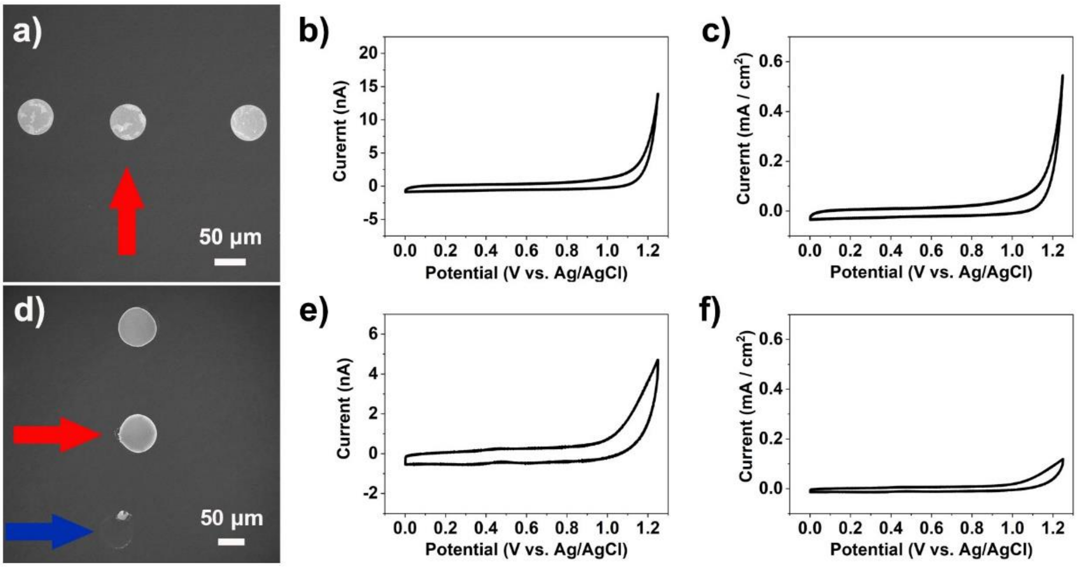

Figure S11. SEM image of the meniscus footprints after experiment, alongside the corresponding CVs and area-normalized CVs on different kind of carbon substrates. **(a-c)** Glassy carbon and **(d-e)** HOPG. The pixels under analysis are indicated by red arrow in both **a)** and **d)**. On HOPG, occasionally little obvious residue was left on the surface, as indicated by blue arrow in **d)**. The CV measurements (v = 1 V s^{-1}) were obtained with probes of diameter *ca.* 70 µm filled with 1 M LiCl solution.

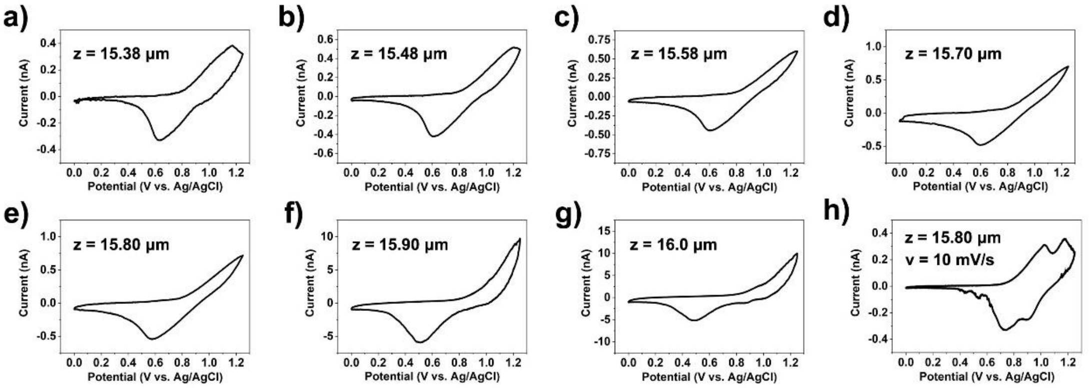

Figure S12. '*Step approach and CV method*' to study the electrochemistry of $LiMn_2O_4$ particles supported on HOPG. From **a)** to **g)** the height is 15.38, 15.48, 15.58, 15.70, 15.80, 15.90, and 16.0 μm, respectively, **h)** was performed at same height with **Figure e)**, but the scan rate was decreased by 100 fold to 10 mV/s. **a)**, **e)**, **g)** and **h)** are reformatted in **Figure 5** in the main text. In **a)**, **b)**, **c)**, **d)**, **e)** and **h)**, the meniscus was not contacted with HOPG, **f)** and **g)** was contacted with HOPG.

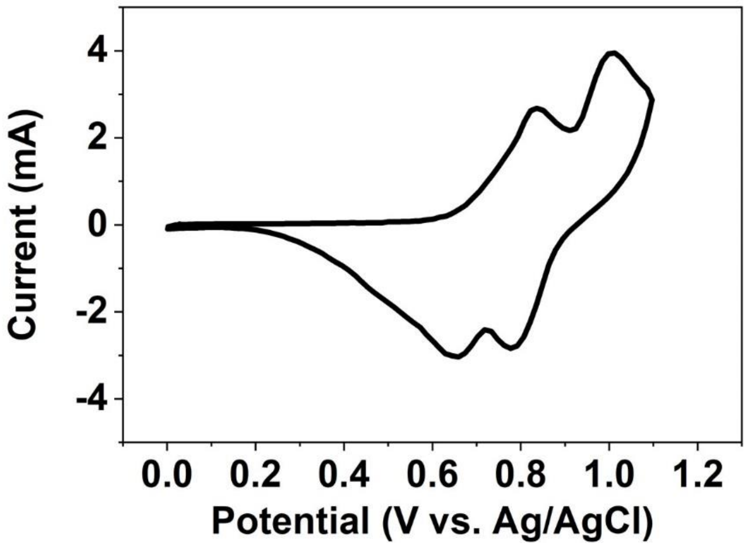

Figure S13. Macroscopic CVs obtained from a composite $LiMn_2O_4$ electrode, at scan rates of 2 mV/s. Two pairs of (de)intercalation peaks can be observed, which is very similar with the result from **Figure 6b** in the main text (or **Figure S12h** above).

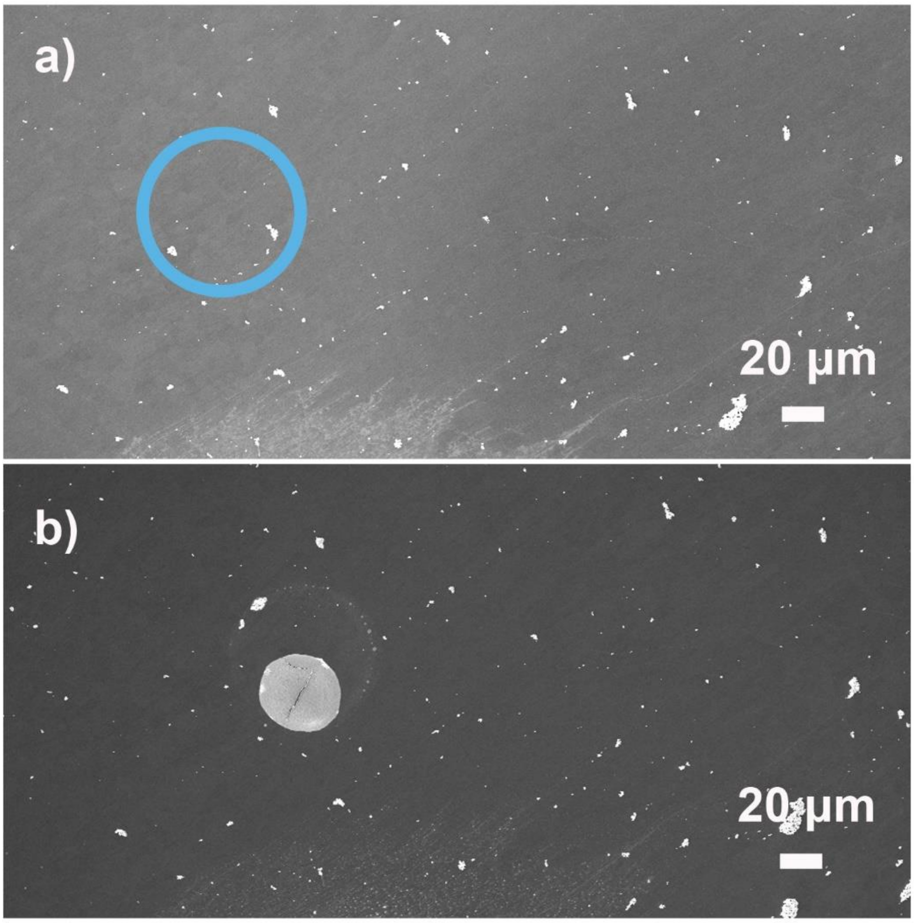

Figure S14. Cyclic voltammetry experiments performed on $LiMn_2O_4$ particles supported HOPG electrode with tips of diameter *ca.* 70 µm. Before **a)** and after **b)** experiment. The probing area is indicated by blue circle in **a)**.

2. Finite Element Method Modeling

Modeling was carried out to understand the key factors controlling the shape of voltammograms recorded under the conditions of the experiments. The model is based on the insertion and transport of Li^+ inside a homogeneous spherical particle supported on an electrode and immersed in a solution of Li^+ ions. The rate of (de)insertion is dependent on the local overpotential at the particle surface, itself a function of both the local potential and the local Li^+ concentration (Figure S15).[1,2] Where mentioned, electronic current is treated using Ohm's law, and is coupled to the Li^+ flux at the particle surface. We note that finite particle conductivity and contact area were neglected in previous models of single particle electrochemistry,[3] but turn out to be essential to reproduce the distorted current-potential behavior observed in our experiments, as compared to the highly regular response observed at much lower scan rates. As a first approximation, only Li^+ transport was considered in the electrolyte, using dilute solution theory. No significant perturbation of the solution Li^+ concentration was observed using this model, meaning solution transport would not be expected to contribute to the observed kinetics.

Throughout the system the concentration of Li^+, c_i, at time, t, is governed by Fick's second law (Equation S1), where the assumption is made that the flux, $\boldsymbol{j_i}$, is solely due to diffusion (Equation S2). This greatly simplifies computation and is subsequently justified by the relatively minor contribution of mass transport to the current (as seen by the small concentration gradients present in the particle). The diffusion coefficients inside $(i = s)$ and outside $(i = l)$ the solid particle were assumed to be constant and independent of concentration. Where included, electronic current, $\boldsymbol{J}$, is treated with Ohm's law (Equation S3) based on either a constant conductivity (σ=10^{-4} S cm^{-1}) or a local composition-dependent conductivity, $\sigma(c)$, the electric field, $\boldsymbol{E}$, (Equation S4) where, ϕ, is the electrostatic potential, and $\boldsymbol{J}_{\text{ext}}$, the current from an external source. Charge is conserved through Equation S5. Initially the conductivity function was based on the arbitrary piecewise cubic interpolation of experimental data (Figure S16), but better correspondence with experimental voltammograms was achieved with a different arbitrary, monotonic conductive function plotted in the in main text (black curve, Figure 7g, inset).

$$\frac{\partial c_i}{\partial t} + \nabla \cdot \boldsymbol{j_i} = 0 \quad \text{(S1)}$$

$$\boldsymbol{j_i} = -D_i \nabla c_i \quad \text{(S2)}$$

$$\boldsymbol{J} = \ -\sigma(c)\boldsymbol{E} + \boldsymbol{J}_{\text{ext}} \quad \text{(S3)}$$

$$\boldsymbol{E} = \nabla \phi \quad \text{(S4)}$$

$$\nabla \cdot \boldsymbol{J} = 0 \quad \text{(S5)}$$

The model was solved for the 2D axisymmetric domain shown in Figure S17, with a particle radius r_0 making contact with radius r_c on an electrically conducting substrate (boundary B1). Transport and Ohm's law were solved for the solid phase, and only transport for the liquid phase. The location of boundary B2 was set to $1.5r_0$ and boundary B3 to $2.5r_0$. The conditions applied at the boundaries are shown in Table S1, where Butler-Vomer kinetics are used to describe the dependence of Li^+ insertion rate on local potential and local Li^+ concentration. The ion flux and electronic current are coupled by the electric current boundary condition on boundary B4 (S11).

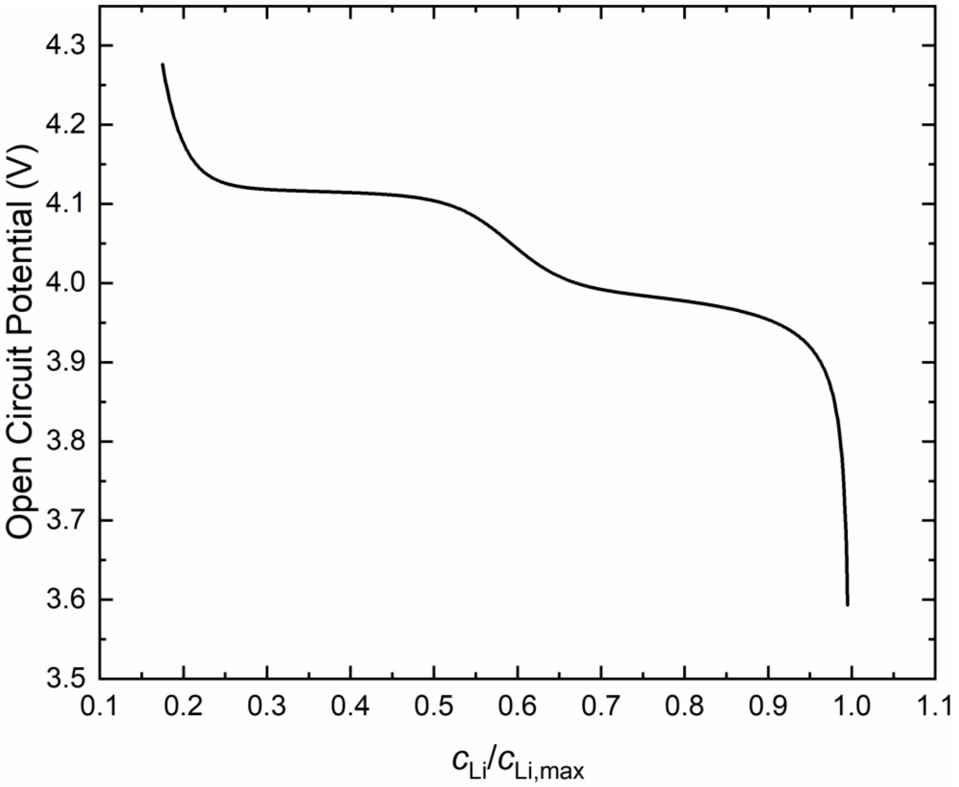

Figure S15. Open circuit potential (OCP)-State of charge (SOC) relationship used in the model.[2]

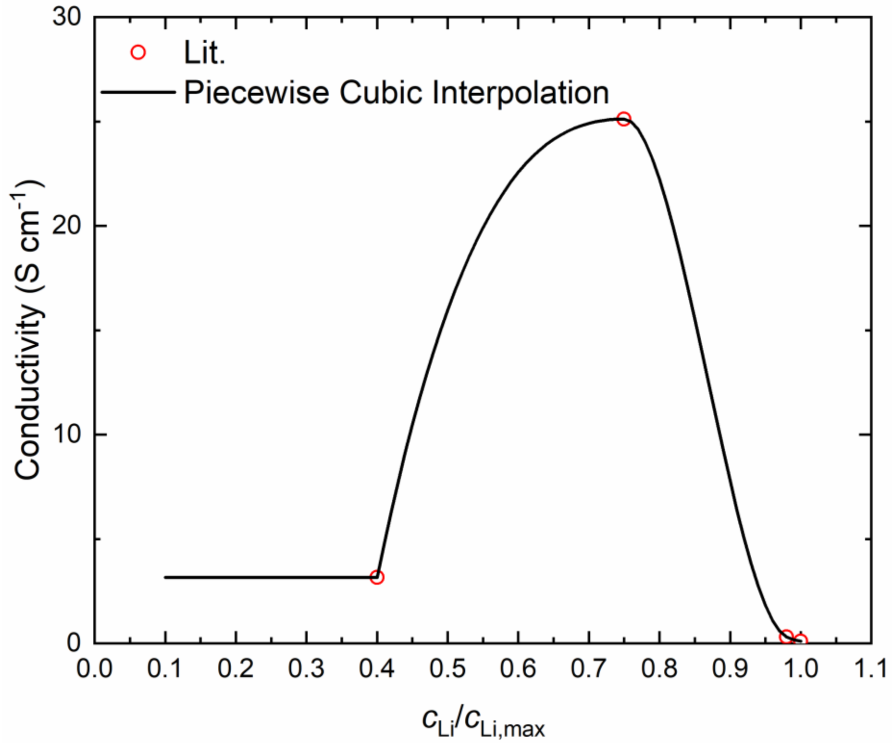

Figure S16. Arbitrary piecewise cubic interpolation of conductivity data.[5]

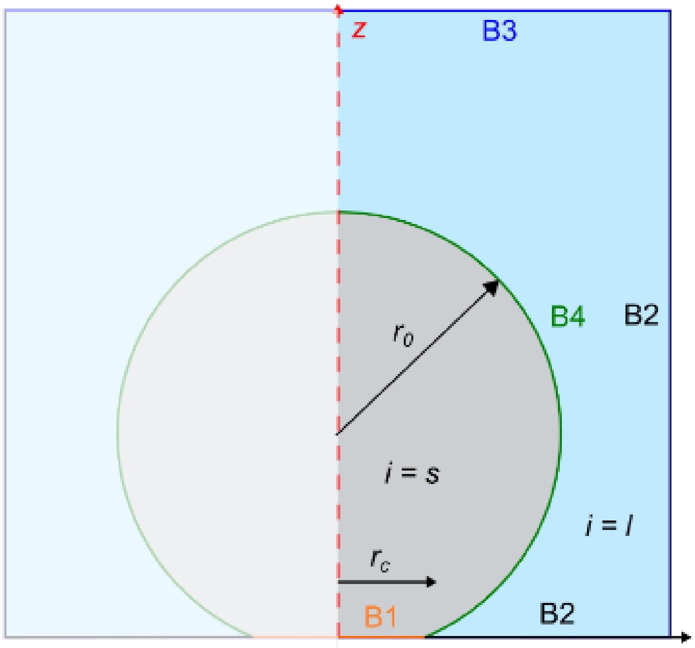

Figure S17. Geometry used for simulations of varying resistance, particle size and particle-substrate contact. Boundaries are labelled B1-B4.

Table S2. Boundary conditions used in the FEM model

	Li⁺ Flux		Electric Current	
B1	$\boldsymbol{n}\cdot\boldsymbol{j_s}=0$	(S6)	$\phi=\phi_{\text{start}}+\nu t$ $(0<t<t_{\text{switch}})$ $\phi=\phi_{\text{switch}}-\nu t$ $(t_{\text{switch}}<t<t_{\text{end}})$	(S7)
B2	$\boldsymbol{n}\cdot\boldsymbol{j_l}=0$	(S8)	-	
B3	$c_l=1\ \ M$	(S9)	-	
B4	$\boldsymbol{j_s}=-\boldsymbol{j_l} = \frac{j_0}{F}\left(\exp\left(\frac{(1-\beta)F}{RT}(\phi-\phi_{\text{OC}}(c_s/c_s^{\max})\right) - \exp\left(\frac{-\beta F}{RT}(\phi-\phi_{\text{OC}}(c_s/c_s^{\max}))\right)\right)$ $j_0=Fk(c_s^{\max}-c_s)^{(1-\beta)}c_s{}^{\boldsymbol{\beta}}c_l^{(1-\beta)}$	(S10)	$\boldsymbol{J}=\boldsymbol{j_s}F$	(S11)

Table S3. Initial conditions used in the FEM model

Domain	Li⁺ Flux	Electric Current
s	$c_s=\ \ 0.995c_s^{\max}$	0
l	$c_l=1\ \ M$	0

Table S4. Parameters used in simulated voltammograms (unless stated otherwise)

Parameter	Meaning	Value	Unit	Ref
β	Symmetry factor	0.5		
F	Faraday's constant	96 485	C mol^{-1}	
R	Molar gas constant	8.314	J K^{-1} mol^{-1}	
T	Temperature	298	K	
$c_s^{\max}$	Li^+ concentration in $LiMn_2O_4$	23.7	mol dm^{-3}	[3]
σ	Electrical conductivity of $LiMn_2O_4$	10^{-4}	S cm^{-1}	[3]
D_s	Diffusion coefficient of Li^+ in $LiMn_2O_4$	2.2×10^{-9}	cm^2 s^{-1}	[3]
D_l	Diffusion coefficient of Li^+ in solution	10^{-5}	cm^2 s^{-1}	
k	Heterogeneous rate constant	10^{-3}	$cm^{5/2}$ s^{-1} $mol^{-1/2}$	
ν	Voltage scan rate	1	V s^{-1}	
r_0	$LiMn_2O_4$ particle radius	100	nm	
r_c	Particle-substrate contact radius	10	nm	

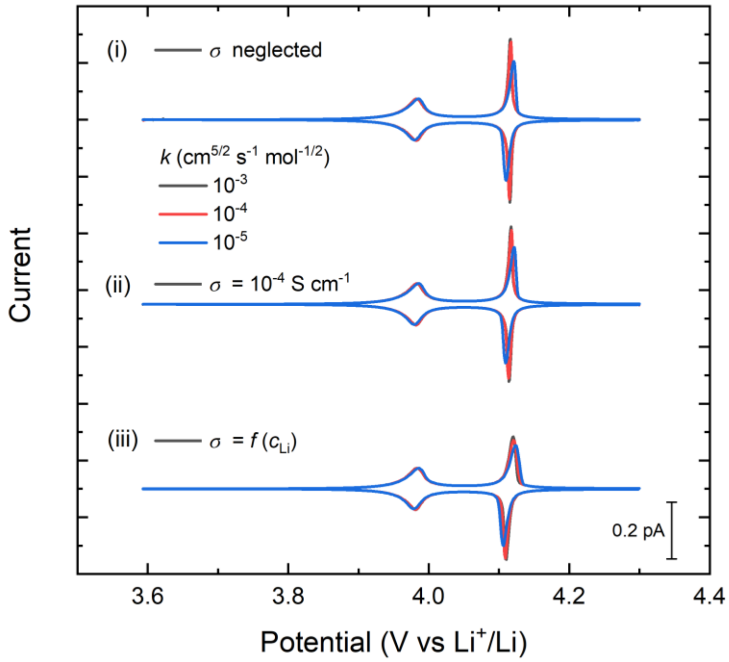

Figure S18. Simulated voltammetry of a single $LiMn_2O_4$ particle at 1 mV s^{-1} with rate constants, k = 10^{-3}, 10^{-4} and 10^{-5} $cm^{5/2}$ s^{-1} $mol^{-1/2}$, for the case of neglected particle conductivity (σ neglected), constant conductivity ($\sigma = 10^{-4}$ S cm^{-1}) and variable conductivity ($\sigma = f(c_{Li})$, function shown in Figure S16).

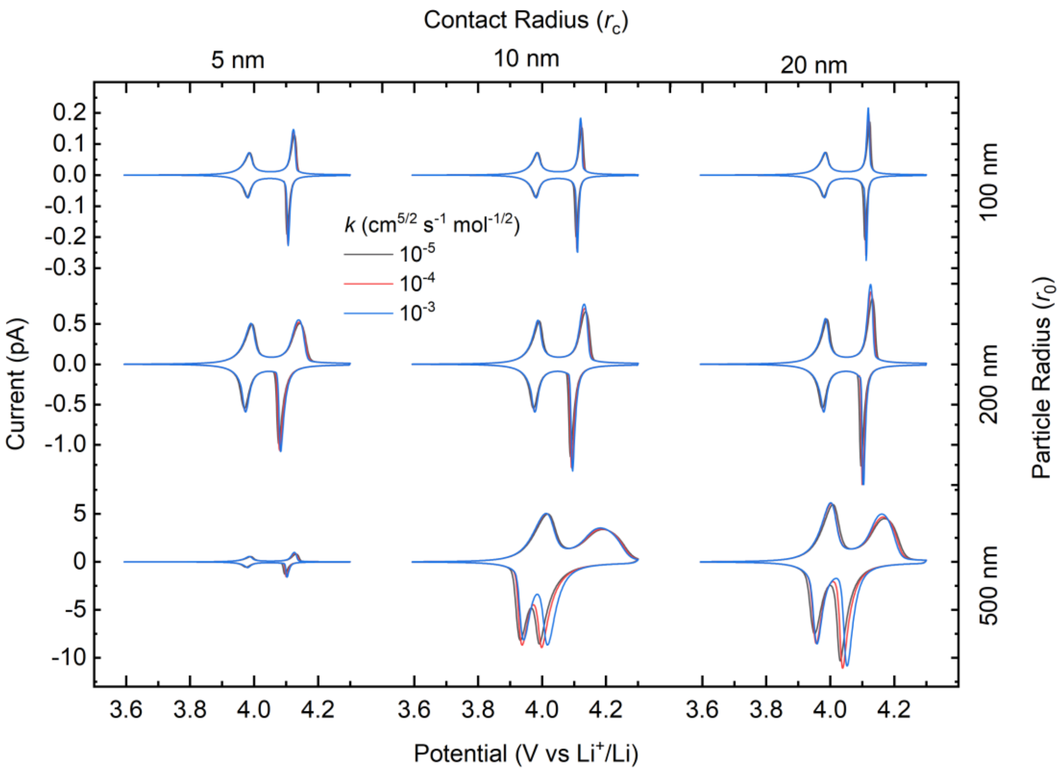

Figure S19. The effect of particle size and contact area on voltammogram shape. Scan rate 1 mV s^{-1}. $\sigma = f(c_{Li})$, the exponential-type SOC-conductivity relationship was used (black curve, Figure 7g, inset).

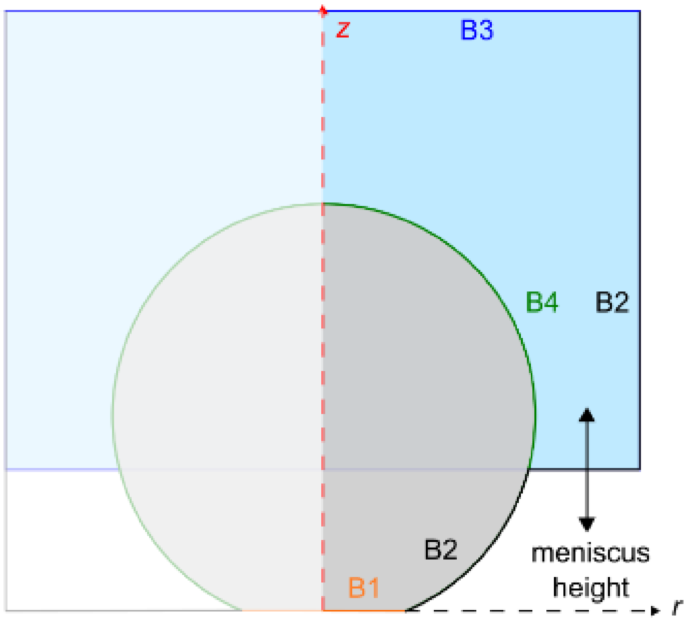

Figure S20. Geometry used to simulate voltammetry with meniscus height varied.

3. References

(1) Doyle, M.; Newman, J. The Use of Mathematical Modeling in the Design of Lithium/Polymer Battery Systems. *Electrochimica Acta* **1995**, *40* (13), 2191–2196. DOI 10.1016/0013-4686(95)00162-8.

(2) Doyle, M.; Newman, J.; Gozdz, A. S.; Schmutz, C. N.; Tarascon, J.-M. Comparison of Modeling Predictions with Experimental Data from Plastic Lithium Ion Cells. *J. Electrochem. Soc.* **1996**, *143* (6), 1890. DOI 10.1149/1.1836921.

(3) Zhang, D.; Popov, B. N.; White, R. E. Modeling Lithium Intercalation of a Single Spinel Particle under Potentiodynamic Control. *J. Electrochem. Soc.* **2000**, *147* (3), 831. DOI 10.1149/1.1393279.

(4) Doyle, M.; Fuller, T. F.; Newman, J. Modeling of Galvanostatic Charge and Discharge of the Lithium/Polymer/Insertion Cell. *J. Electrochem. Soc.* **1993**, *140* (6), 1526–1533. DOI 10.1149/1.2221597.

(5) Park, M.; Zhang, X.; Chung, M.; Less, G. B.; Sastry, A. M. A Review of Conduction Phenomena in Li-Ion Batteries. *Journal of Power Sources* **2010**, *195* (24), 7904–7929. DOI 10.1016/j.jpowsour.2010.06.060.
